# Supplementary material for: New recognition specificity in a plant immune receptor by molecular engineering of its integrated domain
Source: Nat Commun. 2022 Mar 21;13:1524. doi: 10.1038/s41467-022-29196-6 (PMC8938504; doi:10.1038/s41467-022-29196-6)
Supplement: Supplementary file 1 — Supplementary Information [file 41467_2022_29196_MOESM1_ESM.pdf]

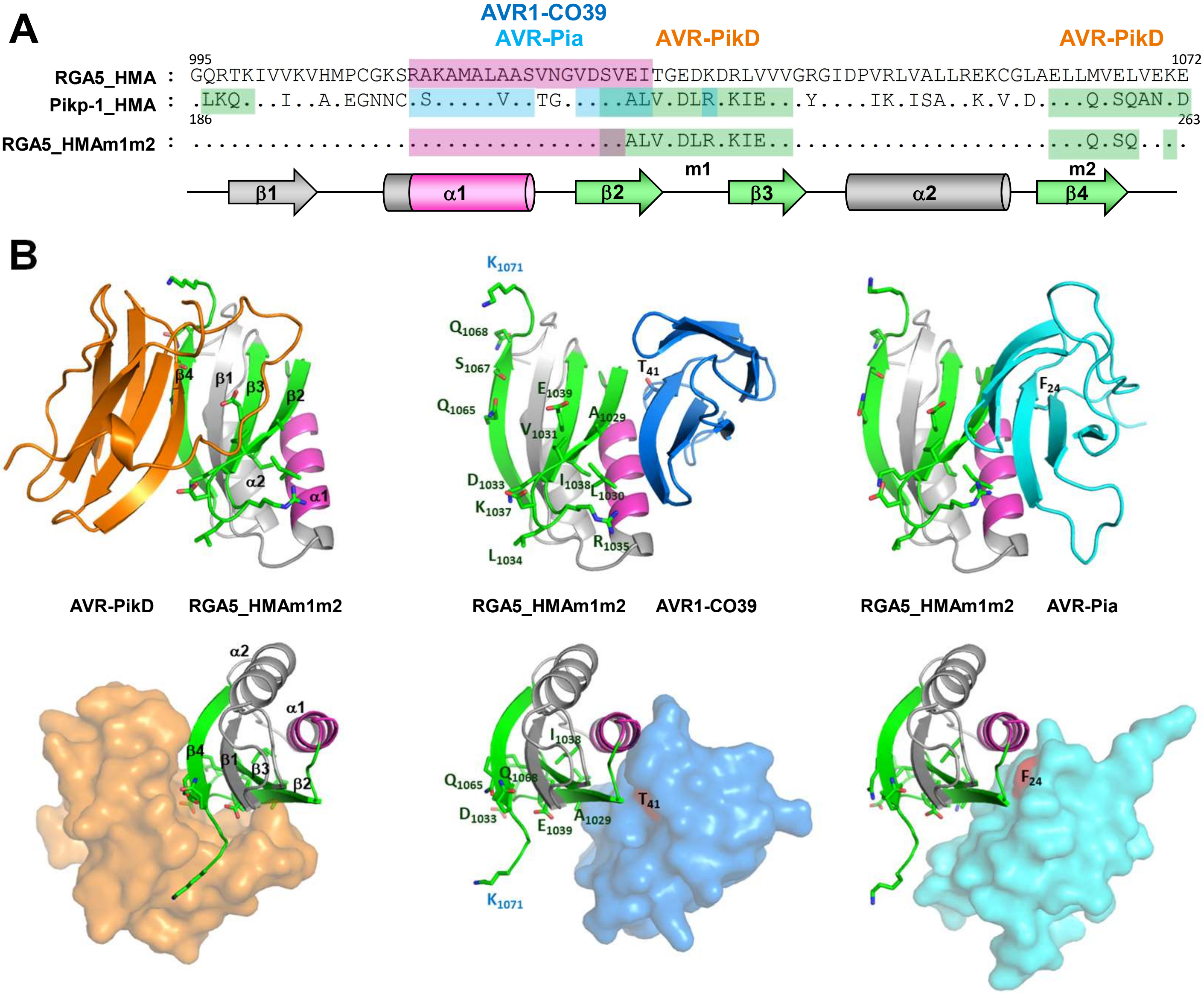

Supplemental figure 1: 3D models of engineered RGA5_HMAm1m2 in complex with MAX effectors. **A)** Sequence alignment of RGA5 and Pikp-1 HMA domains and of the engineered RGA5_HMAm1m2. The residues constituting the binding interface in the complexes AVR1-CO39/RGA5_HMA (PDB_5ZNG), AVR-Pia/Pikp-1_HMA (PDB_6Q76) and AVR-PikD/Pikp-1_HMA (PDB_6G10) are highlighted respectively in pink, blue and green. The peptide fragments comprising strands β2/β3 and β4, targeted respectively by the m1 and m2 mutations and extracted from Pikp-1_HMA crystal structures to construct chimeric 3D models of RGA5_HMAm1m2, are highlighted in green. **B)** Structural models of RGA5_HMAm1m2 in complex with AVR-PikD (orange), AVR1-CO39 (blue) and AVR-Pia (cyan) are shown as cartoons (top views) or molecular surfaces (orthogonal bottom views). The different MAX effector/RGA5_HMAm1m2 complex structures were built by replacing in PDB_5ZNG the effector molecule and the peptide fragments containing the m1 and m2 mutations by their structural counterpart in PDB_6G10 or PDB_6Q76. The secondary structure elements of RGA5_HMAm1m2 in contact with the MAX effectors are colored as in panel (A). Side chains are shown as sticks for the mutated residues in RGA5_HMAm1m2, and for the residues replaced in the inactive effector variants AVR1-CO39_T41G and AVR-Pia_F24S (both highlighted in red in the bottom views) used as negative control in SPR experiments.

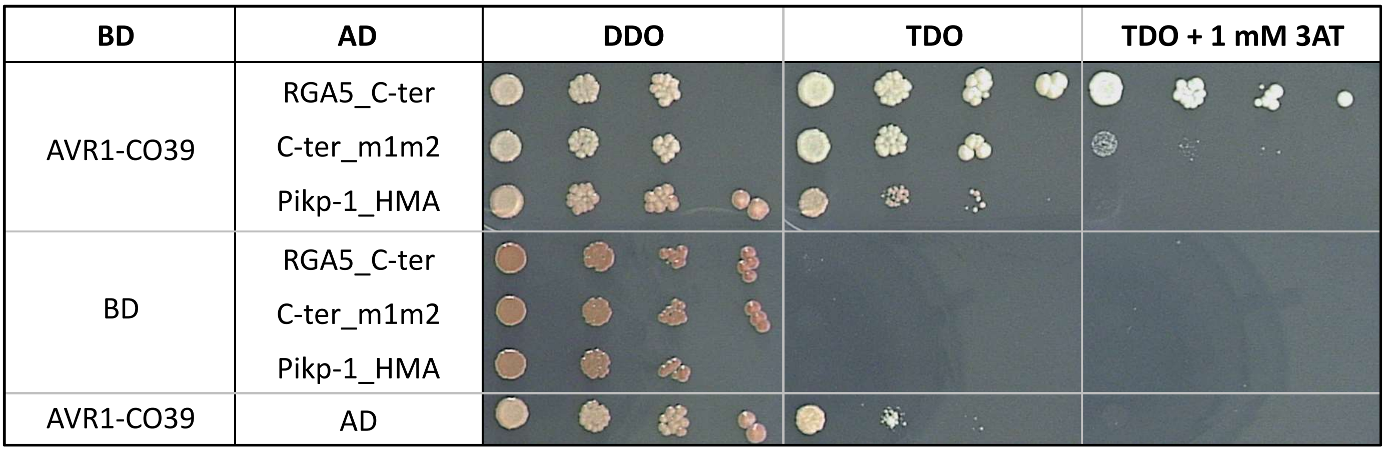

Supplemental figure 2: The m1m2 mutation does not abolish AVR1-CO39 binding to RGA5_C-ter. Interaction of BD-fused AVR1-CO39 (without signal peptides) with the AD-fused C-terminal domains of RGA5 and RGA5m1m2 (residues 883 to 1116) was assayed by yeast two-hybrid experiments. The HMA domain of Pikp-1 (AD:Pikp-1_HMA) and the AD and BD domains of GAL4 were used as controls. Four dilutions of diploid yeast clones (1/1, 1/10, 1/100, 1/1000) were spotted on synthetic TDO (-Trp/-Leu/-His) medium and TDO supplemented with 1 mM of 3-amino-1,2,4-triazole (3AT) to assay for interactions and on synthetic DDO (-Trp/-Leu) to monitor proper growth. Pictures were taken after 5 days of growth.

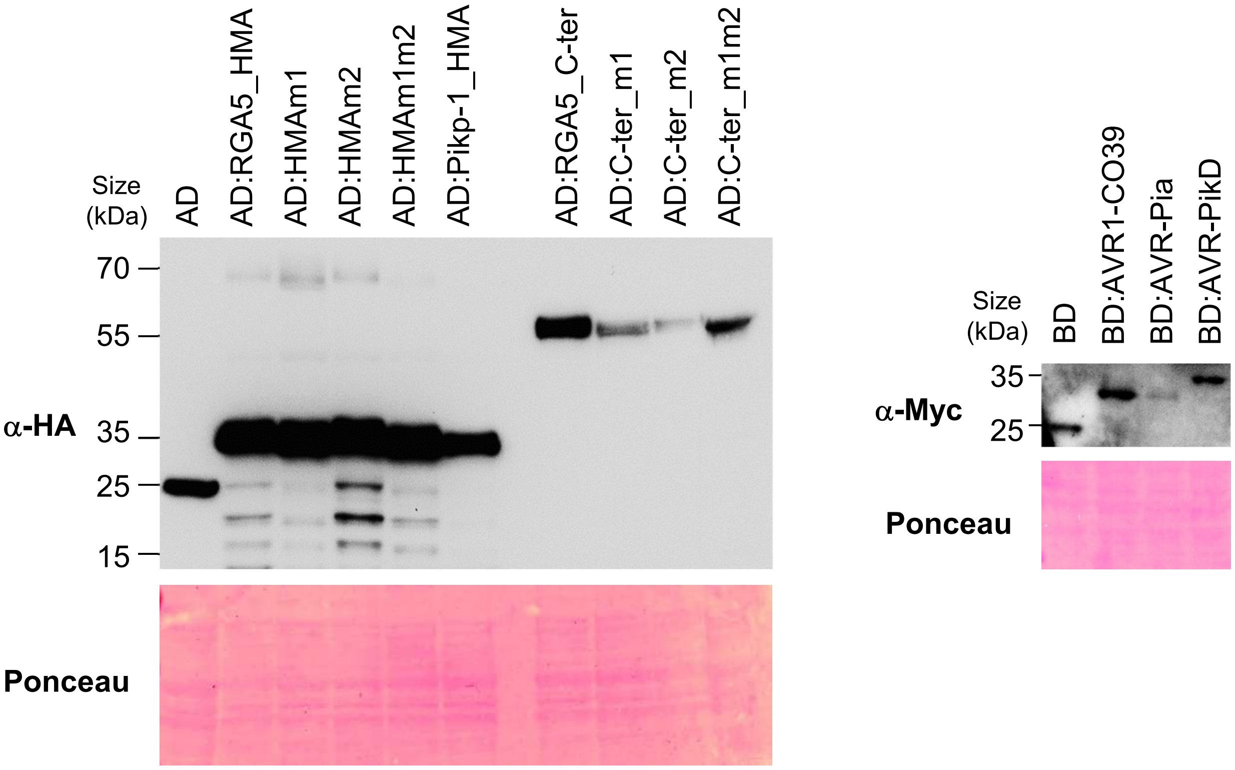

Supplemental figure 3: Presence and integrity of chimeric proteins expressed in yeast. Total proteins were extracted from yeasts and Myc- and HA-tagged proteins were detected by immunoblotting using anti-Myc and anti-HA antibodies, respectively.

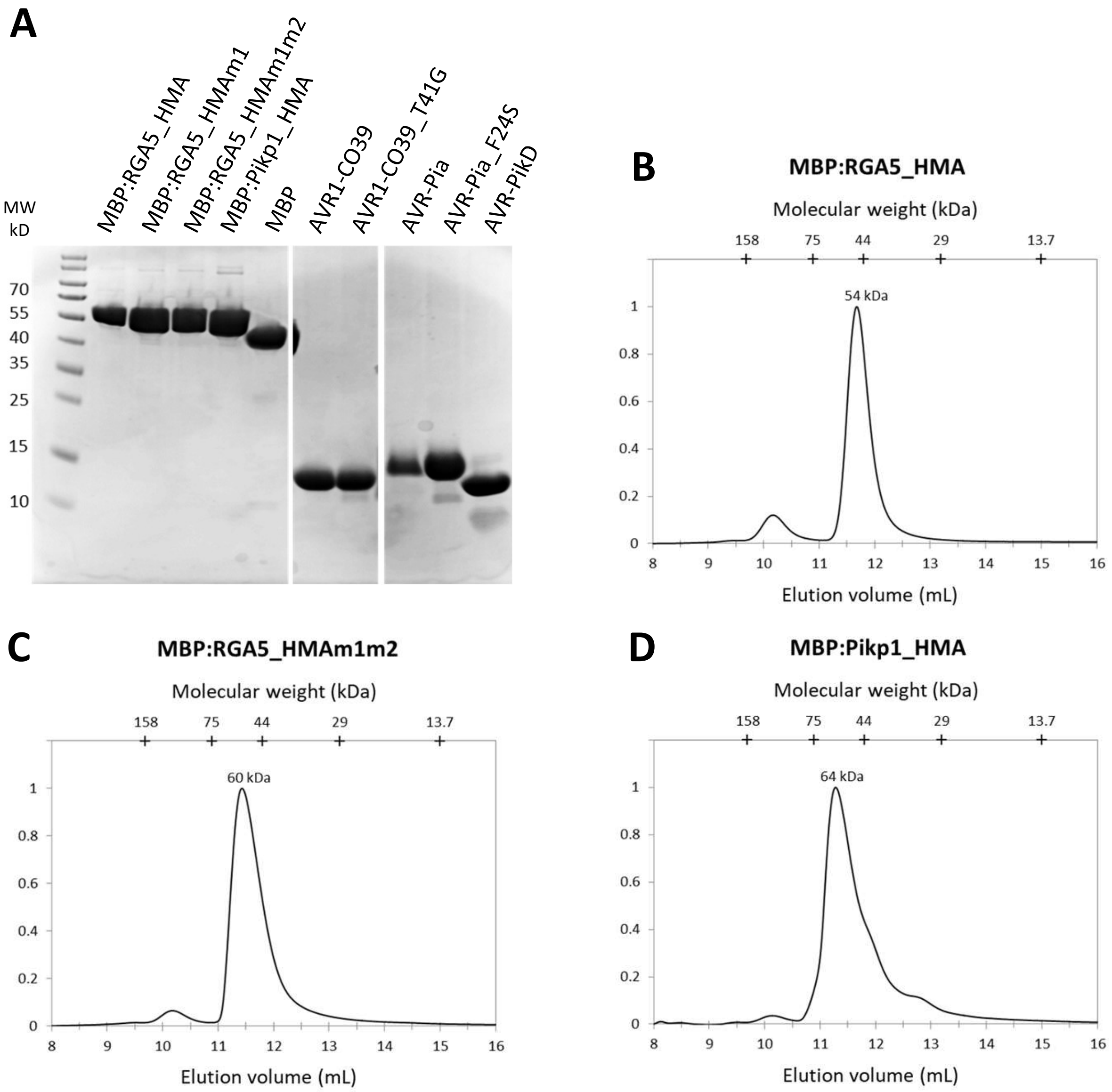

Supplemental Figure 4: Purity and stoichiometry of recombinant proteins. A) SDS-PAGE of the recombinant wild-type and mutant proteins used for SPR analysis. (**B** to **D**) Size exclusion chromatography (SEC) analysis of recombinant HMA domains fused to MBP. 30 µL of the purified proteins at about 2 mg/mL were inject on gel filtration column Superdex 75 10/300 GL (Sigma) at a flow rate of 0.5 mL/min in 20 mM Tris pH 8, 150 mM NaCl and 0.5 mM DTT. Molecular weight (MW) markers (GE healthcare) were used for column calibration (upper horizontal axis) and estimation of the apparent MW of the eluted proteins. Given the theoretical MW of the MBP:HMA protein fusions (52 kDa), these proteins occur mostly as monomers. Wild-type and mutant AVR effectors have been shown to be monomeric (de Guillen et al., 2015, Guo et al., 2018), although AVR1-C039 tends to form dimers in the absence of DTT.

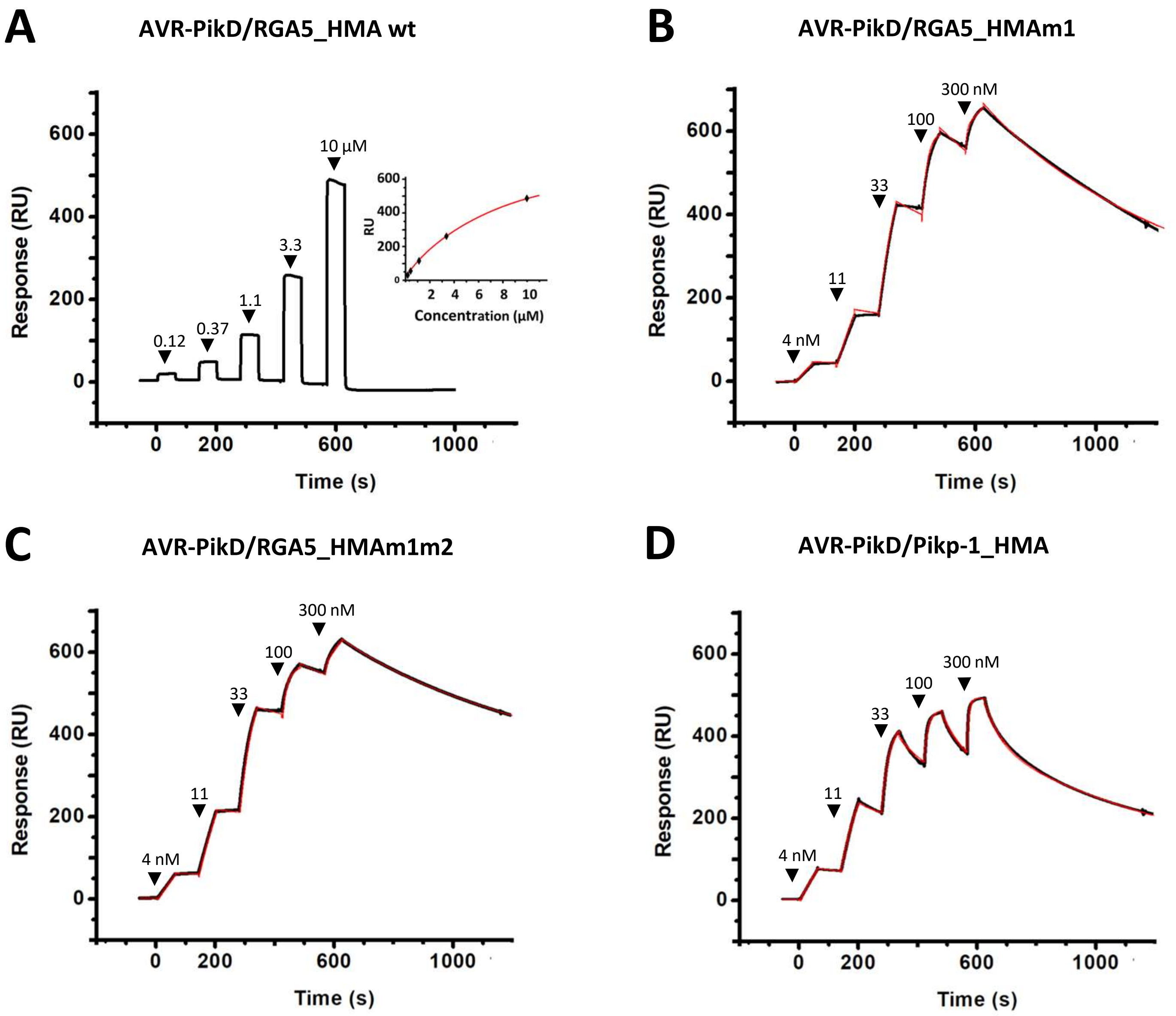

Supplemental figure 5: Single cycle kinetic titrations with AVR-PikD to different MBP:HMAs. The SPR sensorgrams (black curves) show the interaction of the AVR-PikD effector with the different wild-type and mutant HMA domains fused to MBP and captured by anti-MBP antibody immobilized on the chip. Black arrows indicate successive injections of AVR-PikD for 60 sec at the indicated protein concentration, followed by a dissociation phase in running buffer of 80 sec or 600 sec for the final injection. The red curves show the data fit performed by the BiaEvaluation program using a steady-state model (panel A) or a heterogeneous kinetic model (panel B-D). Binding and fitting parameters are reported in Supplemental Table 2.

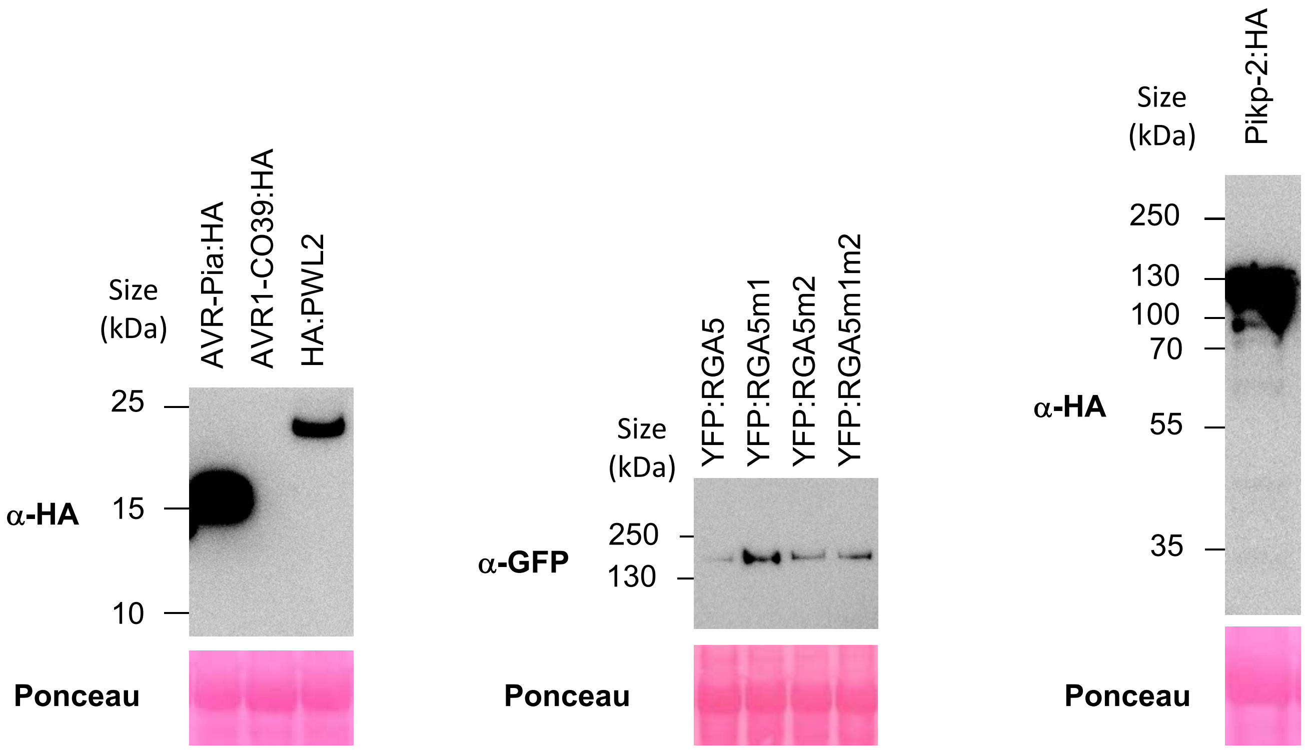

Supplemental figure 6: Presence and integrity of proteins expressed in *N. benthamiana.* Immunoblotting showing expression of HA- and YFP-fused proteins. Total proteins were extracted from transiently transformed *N. benthamiana* leaves 48 h after infiltration and were analyzed by immunoblotting with anti-GFP or anti-HA antibodies. Ponceau staining was used to verify equal protein loading.

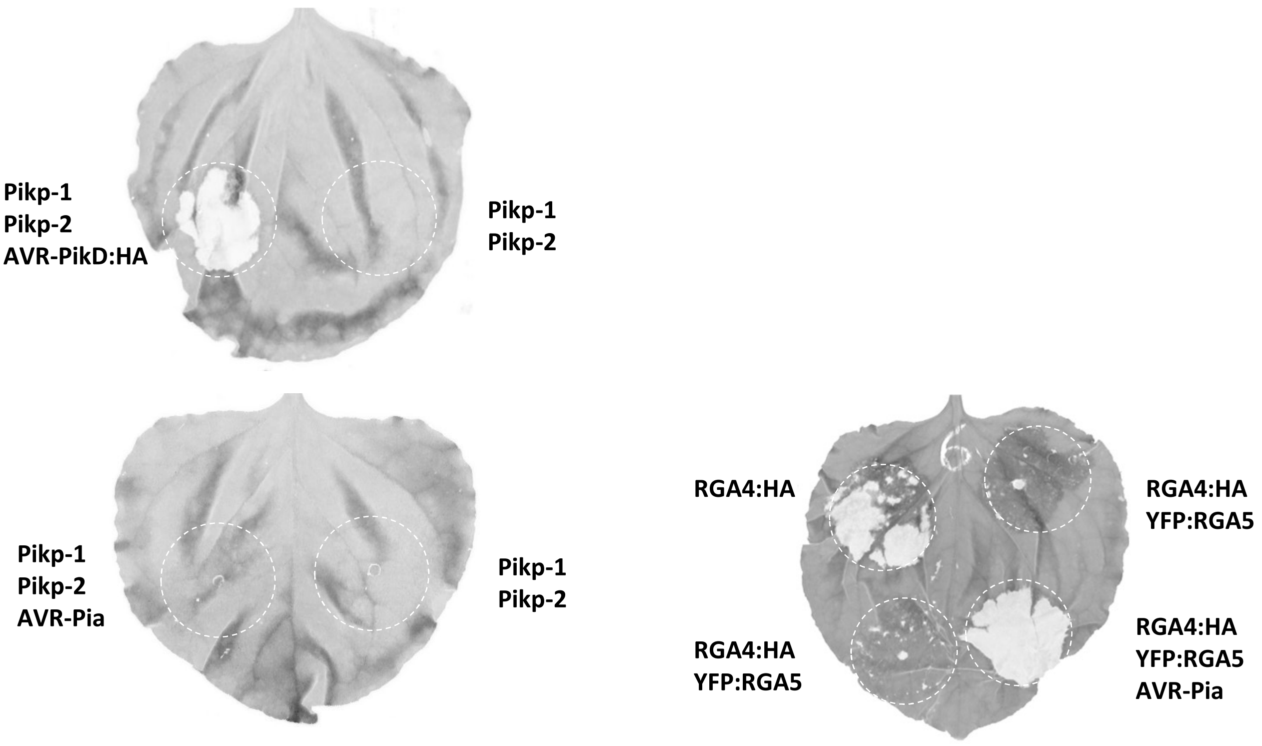

Supplemental figure 7: AVR recognition specificity in *N. benthamiana*. The indicated combinations of constructs were transiently expressed in *N*. *benthamiana* leaves. Cell death was visualized 5 days after infiltration. Greyscale pictures were taken using a fluorescence scanner with settings allowing visualization of cell death (white patches).

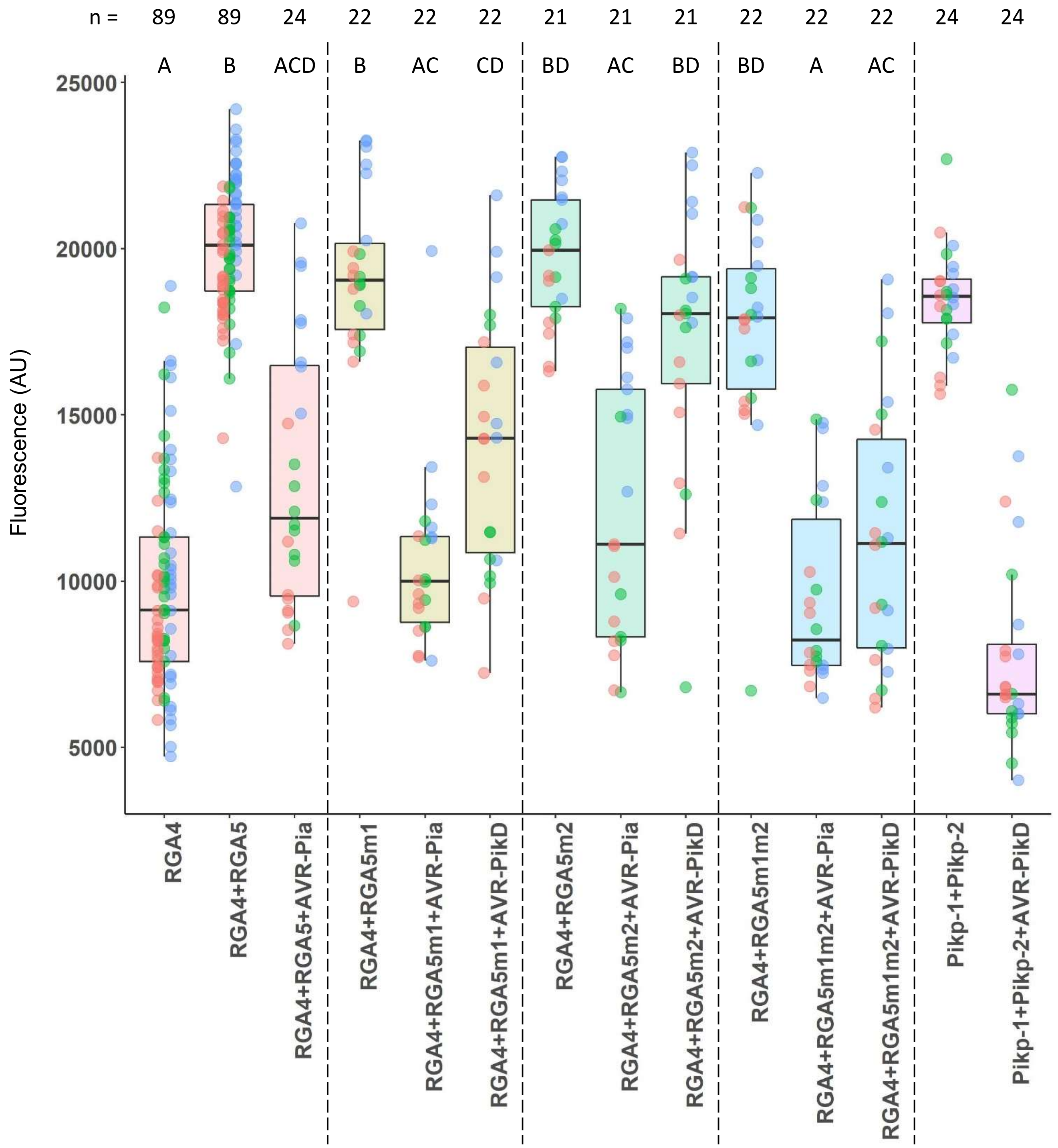

Supplemental figure 8: Recognition of AVR-Pia and AVR-PikD by RGA5m1, m2 and m1m2. The indicated combinations of constructs were transiently expressed in *N. benthamiana* leaves. Greyscale pictures were taken using a fluorescence scanner with settings allowing visualization of the disappearance of red fluorescence due to cell death. Therefore, cell death was quantified by measuring fluorescence levels (arbitrary unit, AU) in the infiltrated areas using ImageJ (Xi et al., 2021). Resulting data were plotted. The boxes represent the first quartile, median, and third quartile. A Kruskal Wallis test followed by a Dunn test were performed to assess difference of fluorescence levels among the various conditions. Groups with the same letter (A to D) are not significantly different at level 0.01. For each combination of constructs, all of the measurements are represented as dots with a distinct color (red, green and blue) for each of the three biological replicates. The sample size (n) for each experimental group is given.

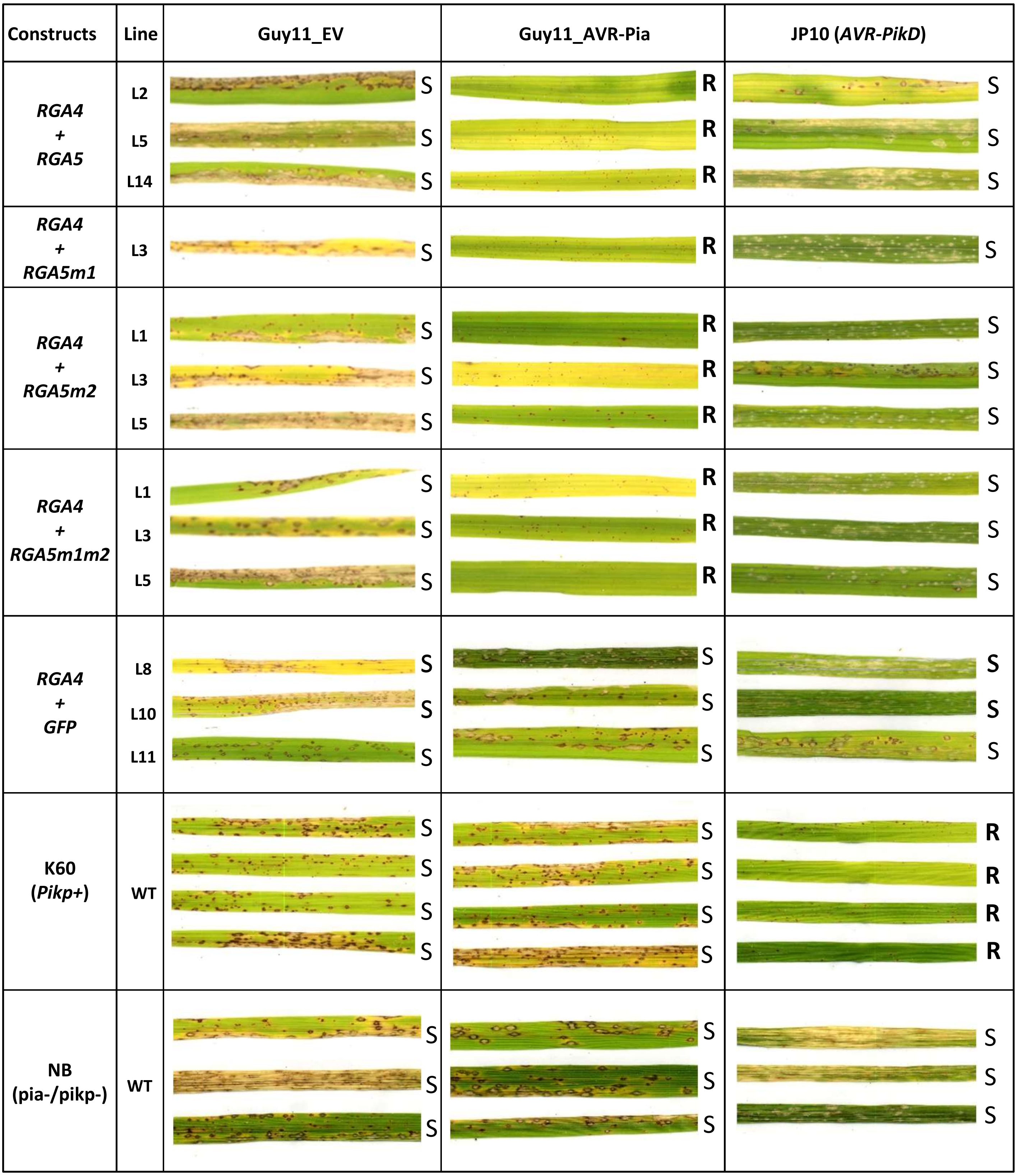

Constructs	Line	Guy11_EV	Guy11_AVR-Pia	JP10 (*AVR-PikD*)
RGA4* + *RGA5	L2	S	R	S
	L5	S	R	S
	L14	S	R	S
RGA4* + *RGA5m1	L3	S	R	S
RGA4* + *RGA5m2	L1	S	R	S
	L3	S	R	S
	L5	S	R	S
RGA4* + *RGA5m1m2	L1	S	R	S
	L3	S	R	S
	L5	S	R	S
RGA4* + *GFP	L8	S	S	S
	L10	S	S	S
	L11	S	S	S
**K60 (*Pikp+*)**	WT	S	S	R
		S	S	R
		S	S	R
		S	S	R
**NB (pia-/pikp-)**	WT	S	S	S
		S	S	S
		S	S	S

Supplemental figure 9: Inoculation of T0 transgenic plants with *M. oryzae*. The rice cultivar Nipponbare was co-transformed with a genomic construct for *RGA4* and a genomic construct for *RGA5*, *RGA5m1*, *RGA5m2* or *RGA5m1m2*. A transgenic line carrying *RGA4* and the *GFP* was also generated as a control. T0 plants of the transgenic lines were spray inoculated with the transgenic strain Guy11-AVR-Pia or the wild-type JP10 (*AVR-PikD*+) isolate. The rice cultivar K60 carrying the *Pikp* resistance was used as a control for AVR-PikD specific recognition while Nipponbare (*pikp-/pia-*) served as negative control. Pictures show representative symptoms at 7 days after inoculation. Individual leaves indicate independent T1 transgenic lines (see Supplemental Table 3). S = susceptible, R = resistant.

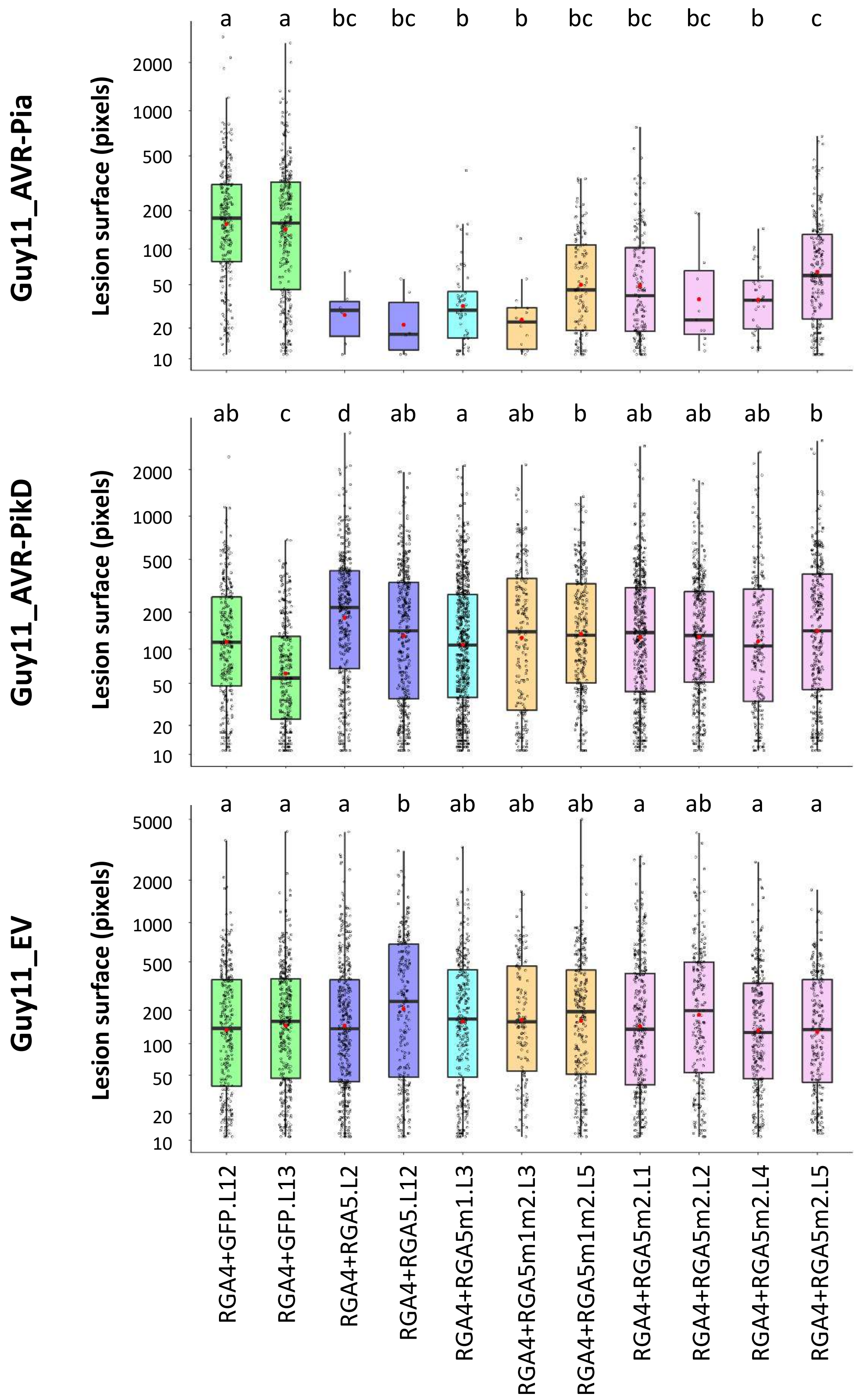

Supplemental figure 10: Disease lesion measurements after inoculation of T2 transgenic plants. Transgenic *M. oryzae* isolates carrying *AVR-Pia*, *AVR-PikD* or the empty vector (EV), were spray-inoculated on T2 transgenic plants carrying the indicated transgenes (i.e. *RGA4+GFP*, *RGA4+RGA5, RGA4 +RGA5m1, RGA4+RGA5m1m2* or *RGA4+RGA5m2*). For each combination of transgenes, the rice transgenic lines used for inoculation are indicated (see Suppl. Table 3). Leaves from 5 to 8 different plants for each transgenic line were scanned 7 days after inoculation. Areas of disease lesions were measured using LeAFtool (https://github.com/sravel/LeAFtool) and plotted. The boxes represent the first quartile, median, and third quartile. Difference of lesion areas among the transgenic lines was assessed by a Kruskall-Wallis test followed by a Dunn test. For each isolate inoculated, groups with the same letter (A to C or D) are not significantly different at level 0.01.

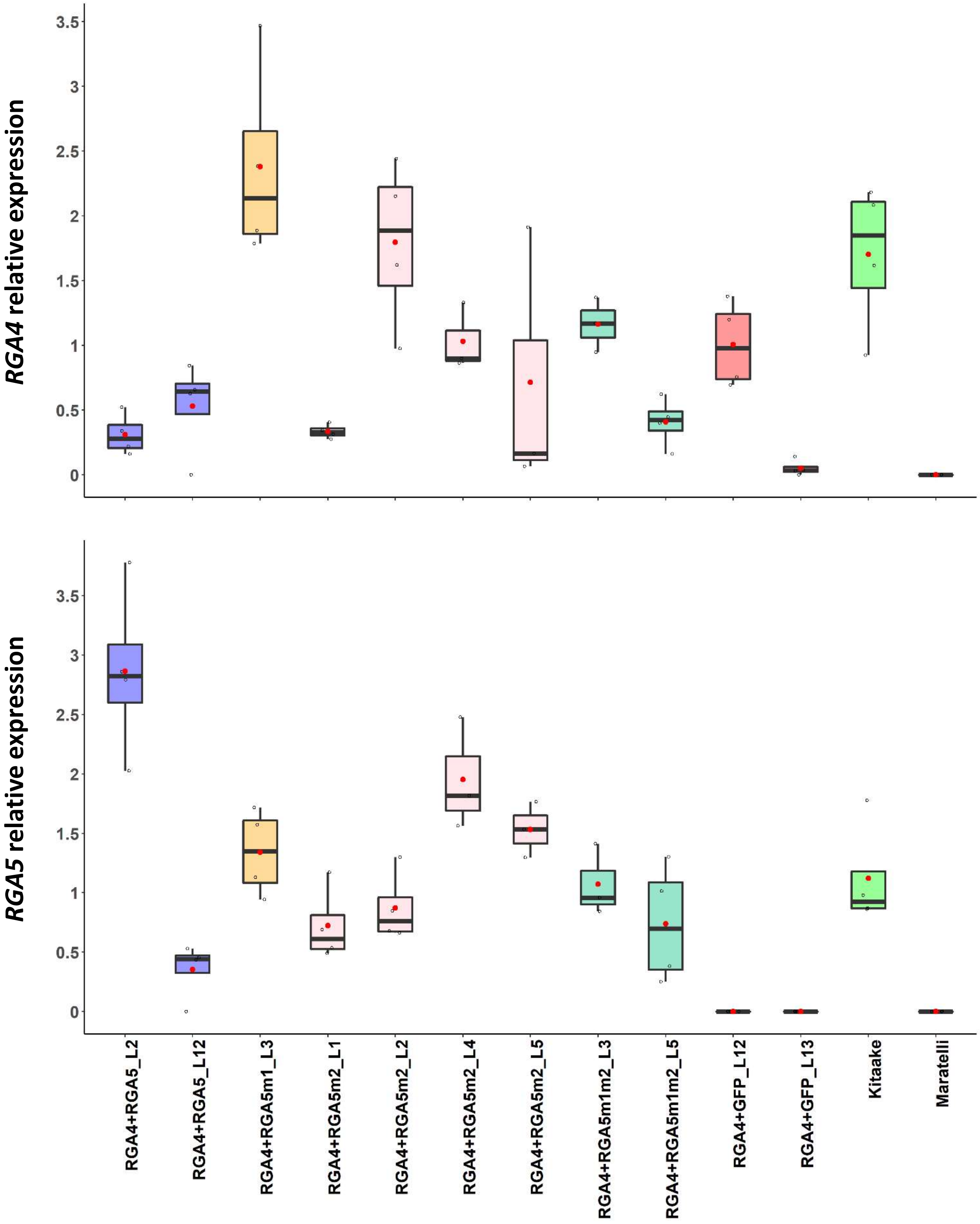

Supplemental Figure 11: Relative expression of RGA4 and RGA5 in rice transgenic lines. mRNA levels of *RGA4* and *RGA5* in the independent rice transgenic lines (L) and in wild type Maratelli (*pia-*) and Kitaake (*Pia+*) were determined by qRT-PCR. Relative expression levels were calculated using the constitutively expressed rice actin gene (*Os03g50890*) as a reference. Red dots indicate the mean values calculated from 3 to 4 plant pools (each pool containing RNA from 3 individual plants) for each transgenic line.

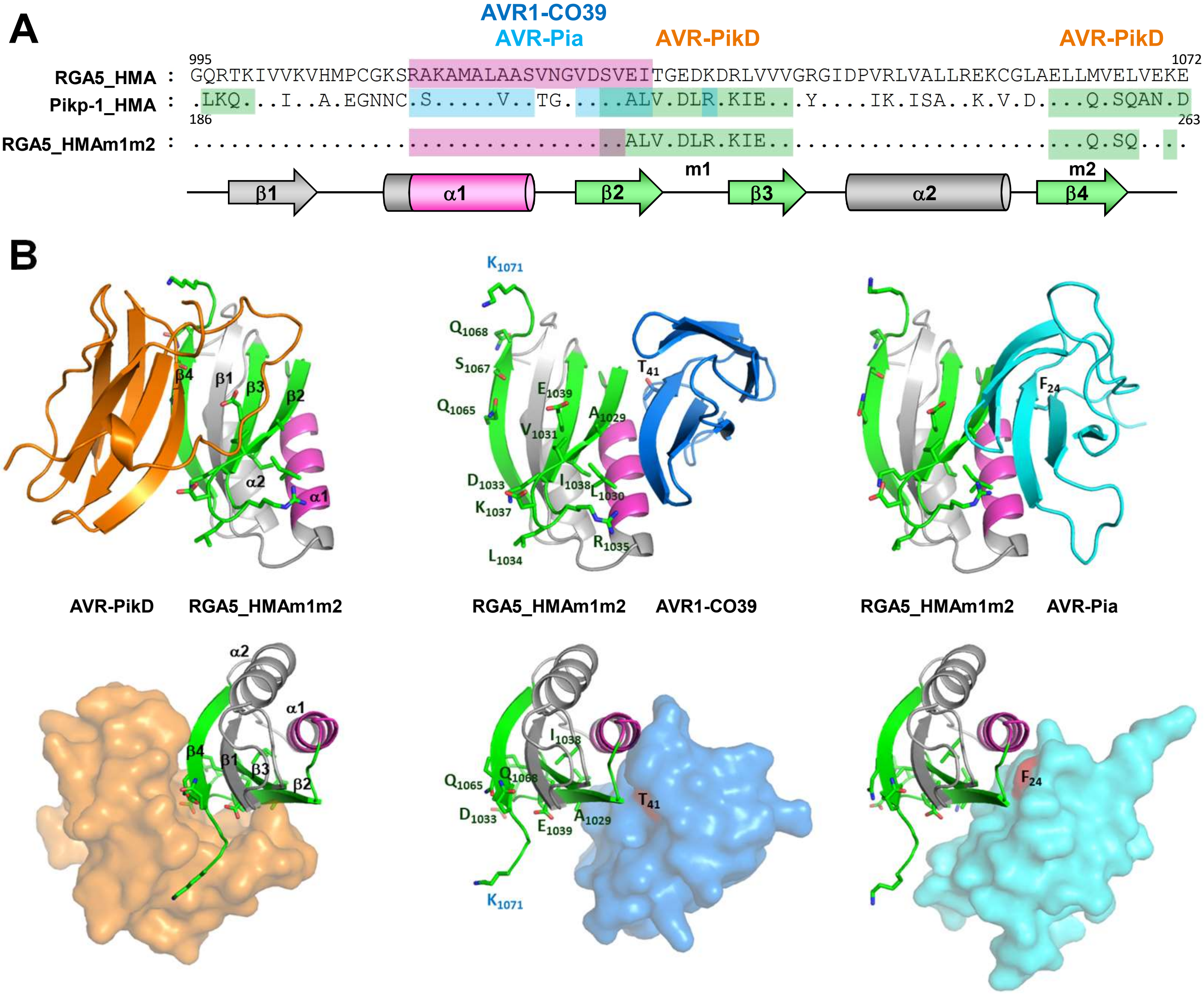

Supplemental figure 1: 3D models of engineered RGA5_HMAm1m2 in complex with MAX effectors. **A)** Sequence alignment of RGA5 and Pikp-1 HMA domains and of the engineered RGA5_HMAm1m2. The residues constituting the binding interface in the complexes AVR1-CO39/RGA5_HMA (PDB_5ZNG), AVR-Pia/Pikp-1_HMA (PDB_6Q76) and AVR-PikD/Pikp-1_HMA (PDB_6G10) are highlighted respectively in pink, blue and green. The peptide fragments comprising strands β2/β3 and β4, targeted respectively by the m1 and m2 mutations and extracted from Pikp-1_HMA crystal structures to construct chimeric 3D models of RGA5_HMAm1m2, are highlighted in green. **B)** Structural models of RGA5_HMAm1m2 in complex with AVR-PikD (orange), AVR1-CO39 (blue) and AVR-Pia (cyan) are shown as cartoons (top views) or molecular surfaces (orthogonal bottom views). The different MAX effector/RGA5_HMAm1m2 complex structures were built by replacing in PDB_5ZNG the effector molecule and the peptide fragments containing the m1 and m2 mutations by their structural counterpart in PDB_6G10 or PDB_6Q76. The secondary structure elements of RGA5_HMAm1m2 in contact with the MAX effectors are colored as in panel (A). Side chains are shown as sticks for the mutated residues in RGA5_HMAm1m2, and for the residues replaced in the inactive effector variants AVR1-CO39_T41G and AVR-Pia_F24S (both highlighted in red in the bottom views) used as negative control in SPR experiments.

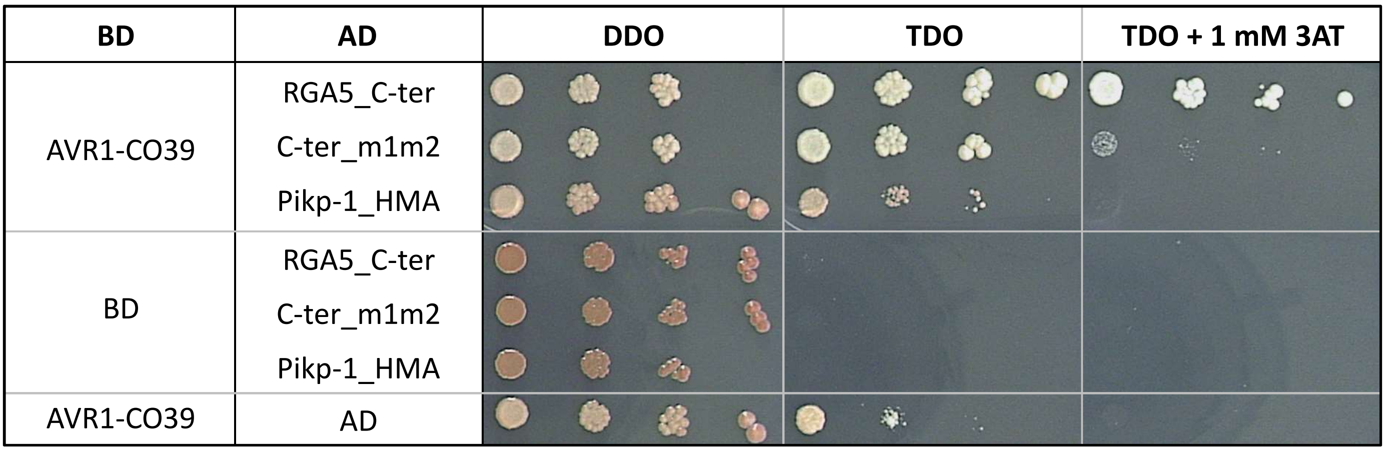

Supplemental figure 2: The m1m2 mutation does not abolish AVR1-CO39 binding to RGA5_C-ter. Interaction of BD-fused AVR1-CO39 (without signal peptides) with the AD-fused C-terminal domains of RGA5 and RGA5m1m2 (residues 883 to 1116) was assayed by yeast two-hybrid experiments. The HMA domain of Pikp-1 (AD:Pikp-1_HMA) and the AD and BD domains of GAL4 were used as controls. Four dilutions of diploid yeast clones (1/1, 1/10, 1/100, 1/1000) were spotted on synthetic TDO (-Trp/-Leu/-His) medium and TDO supplemented with 1 mM of 3-amino-1,2,4-triazole (3AT) to assay for interactions and on synthetic DDO (-Trp/-Leu) to monitor proper growth. Pictures were taken after 5 days of growth.

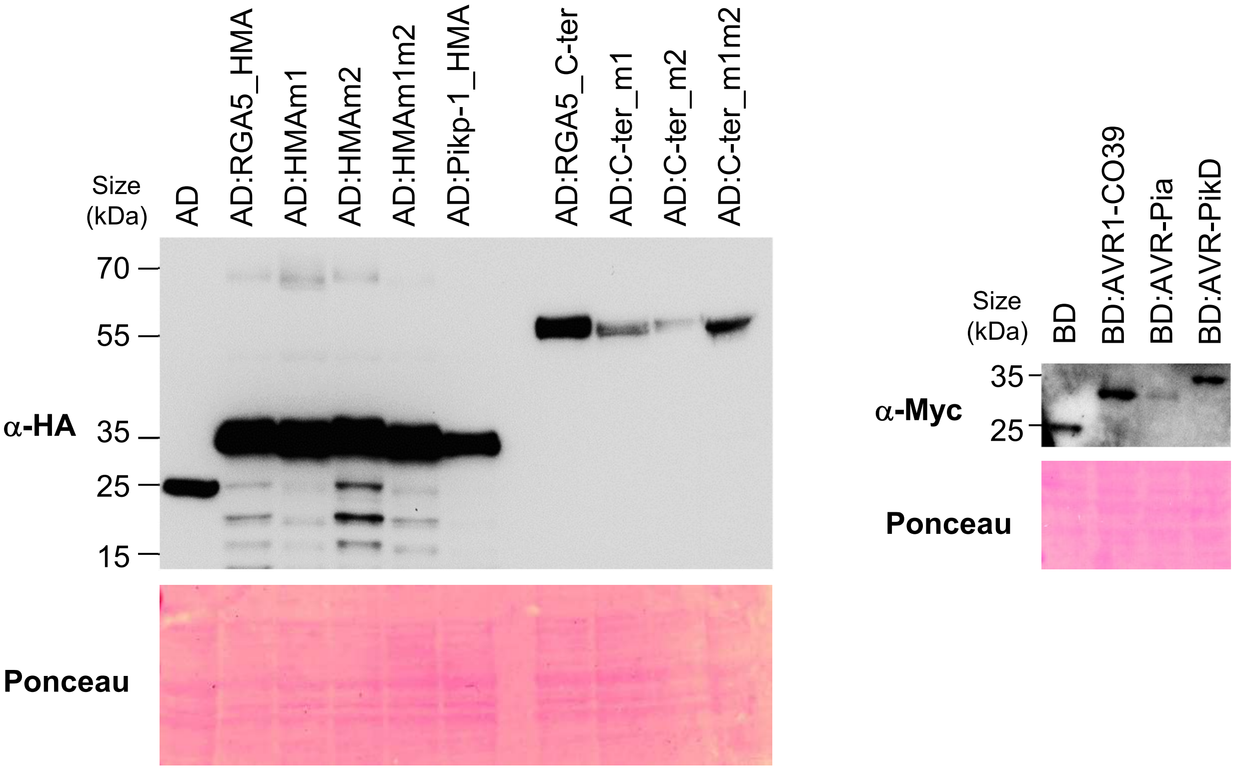

Supplemental figure 3: Presence and integrity of chimeric proteins expressed in yeast. Total proteins were extracted from yeasts and Myc- and HA-tagged proteins were detected by immunoblotting using anti-Myc and anti-HA antibodies, respectively. The experiment was carried out once.

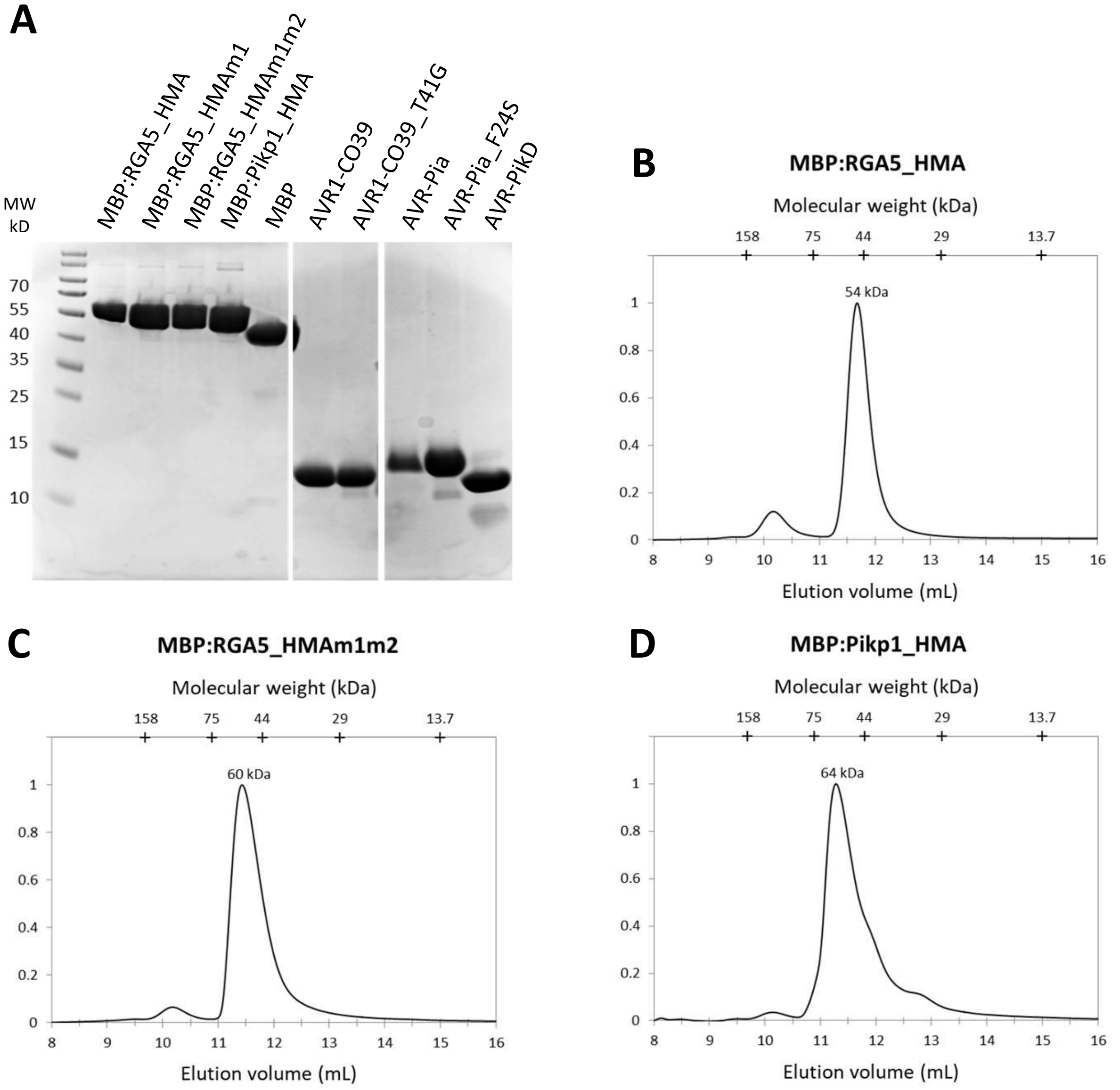

Supplemental Figure 4: Purity and stoichiometry of recombinant proteins. A) SDS-PAGE of the recombinant wild-type and mutant proteins used for SPR analysis. (**B** to **D**) Size exclusion chromatography (SEC) analysis of recombinant HMA domains fused to MBP. 30 µL of the purified proteins at about 2 mg/mL were inject on gel filtration column Superdex 75 10/300 GL (Sigma) at a flow rate of 0.5 mL/min in 20 mM Tris pH 8, 150 mM NaCl and 0.5 mM DTT. Molecular weight (MW) markers (GE healthcare) were used for column calibration (upper horizontal axis) and estimation of the apparent MW of the eluted proteins. Given the theoretical MW of the MBP:HMA protein fusions (52 kDa), these proteins occur mostly as monomers. Wild-type and mutant AVR effectors have been shown to be monomeric (de Guillen et al., 2015, Guo et al., 2018), although AVR1-C039 tends to form dimers in the absence of DTT.

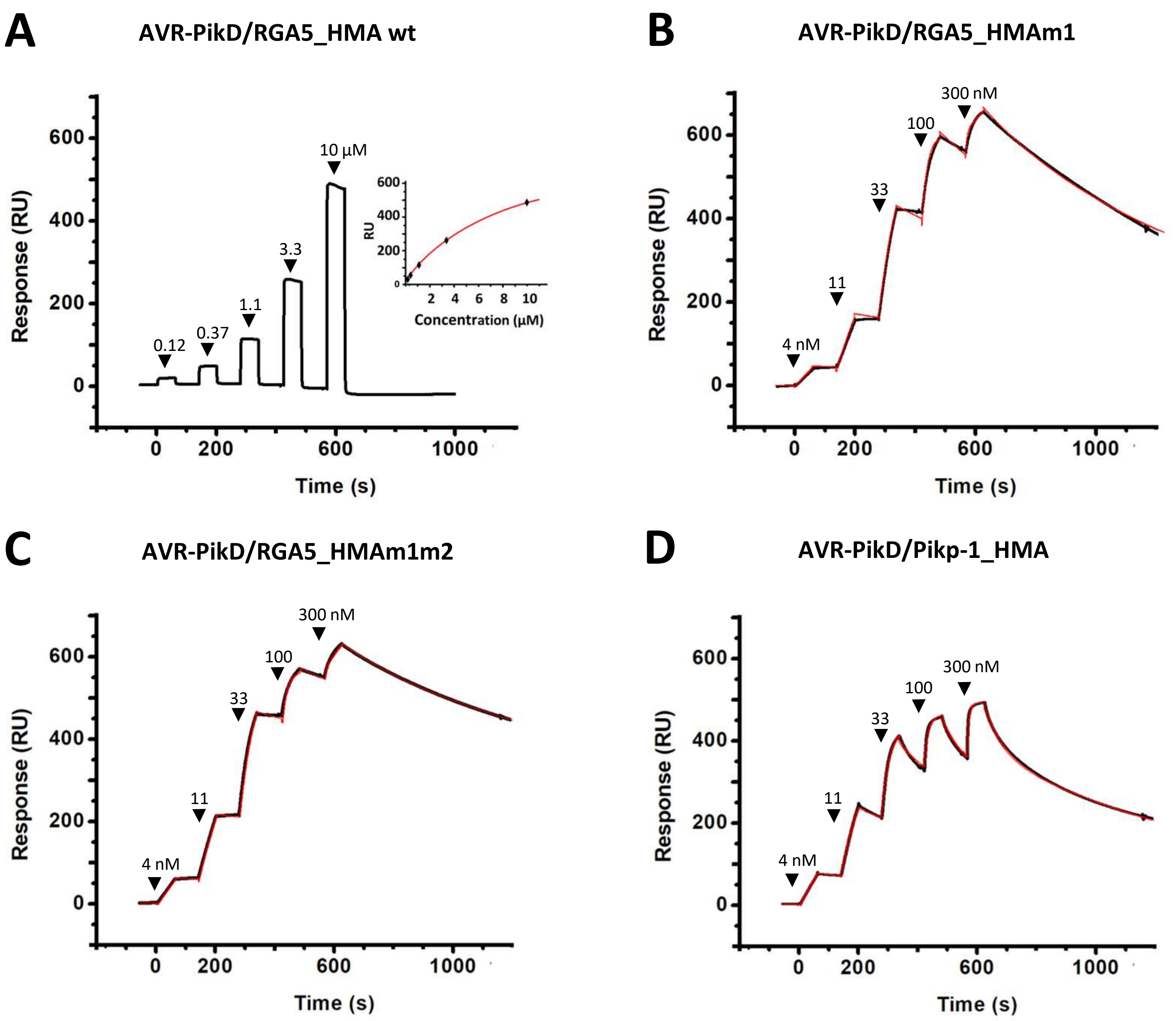

Supplemental figure 5: Single cycle kinetic titrations with AVR-PikD to different MBP:HMAs. The SPR sensorgrams (black curves) show the interaction of the AVR-PikD effector with the different wild-type and mutant HMA domains fused to MBP and captured by anti-MBP antibody immobilized on the chip. Black arrows indicate successive injections of AVR-PikD for 60 sec at the indicated protein concentration, followed by a dissociation phase in running buffer of 80 sec or 600 sec for the final injection. The red curves show the data fit performed by the BiaEvaluation program using a steady-state model (panel A) or a heterogeneous kinetic model (panel B-D). Binding and fitting parameters are reported in Supplemental Table 2.

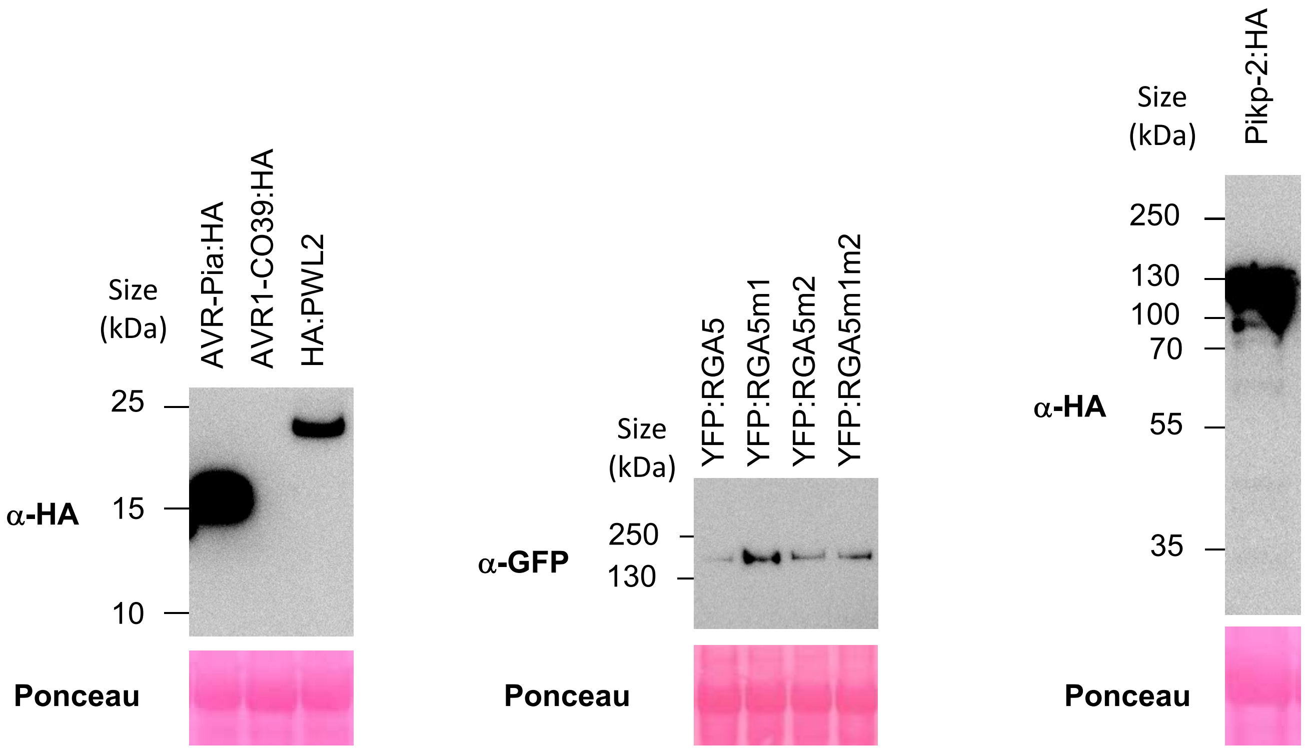

Supplemental figure 6: Presence and integrity of proteins expressed in *N. benthamiana.* Immunoblotting showing expression of HA- and YFP-fused proteins. Total proteins were extracted from transiently transformed *N. benthamiana* leaves 48 h after infiltration and were analyzed by immunoblotting with anti-GFP or anti-HA antibodies. Ponceau staining was used to verify equal protein loading. All immunoblots were performed once.

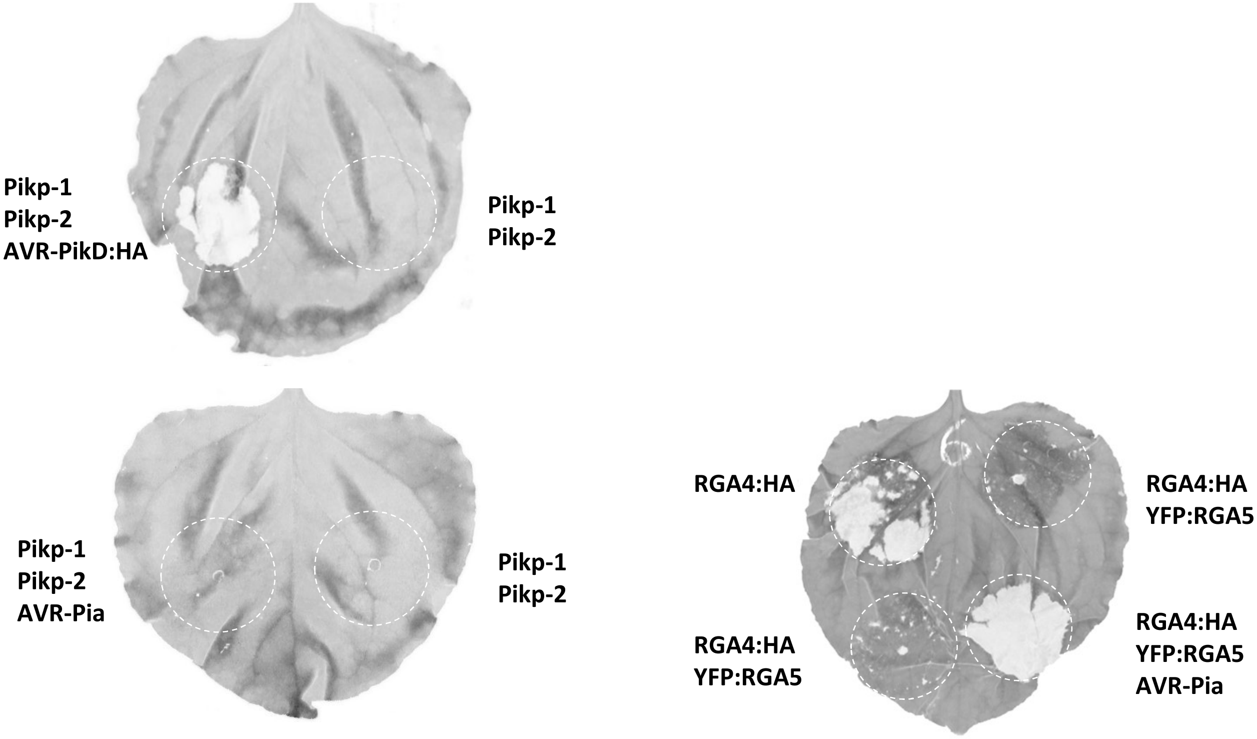

Supplemental figure 7: AVR recognition specificity in *N. benthamiana*. The indicated combinations of constructs were transiently expressed in *N*. *benthamiana* leaves. Cell death was visualized 5 days after infiltration. Greyscale pictures were taken using a fluorescence scanner with settings allowing visualization of cell death (white patches).

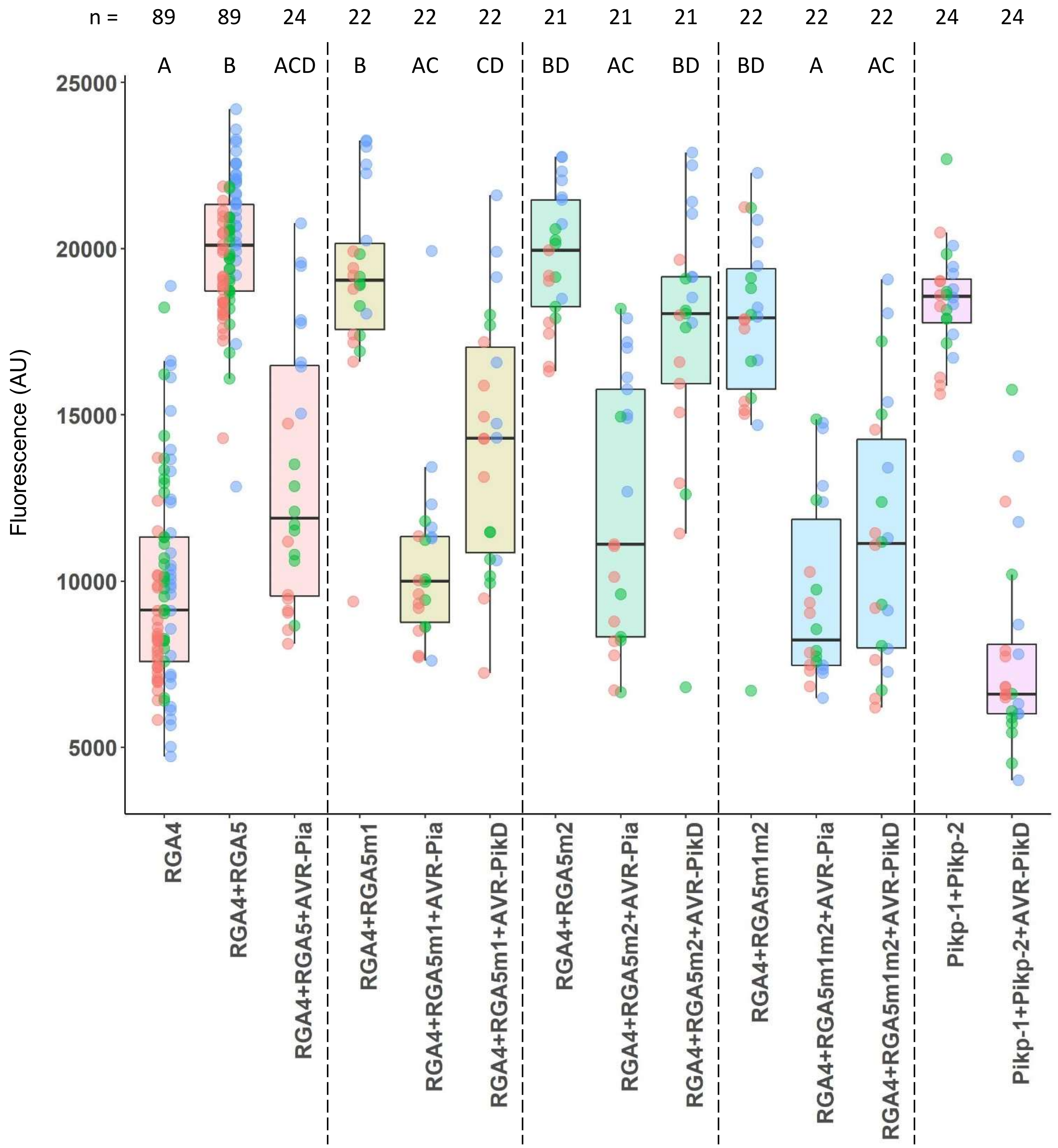

Supplemental figure 8: Recognition of AVR-Pia and AVR-PikD by RGA5m1, m2 and m1m2. The indicated combinations of constructs were transiently expressed in *N. benthamiana* leaves. Greyscale pictures were taken using a fluorescence scanner with settings allowing visualization of the disappearance of red fluorescence due to cell death. Therefore, cell death was quantified by measuring fluorescence levels (arbitrary unit, AU) in the infiltrated areas using ImageJ (Xi et al., 2021). Resulting data were plotted. The boxes represent the first quartile, median, and third quartile. A Kruskal Wallis test followed by a Dunn test were performed to assess difference of fluorescence levels among the various conditions. Groups with the same letter (A to D) are not significantly different at level 0.01. For each combination of constructs, all of the measurements are represented as dots with a distinct color (red, green and blue) for each of the three biological replicates. The sample size (n) for each experimental group is given.

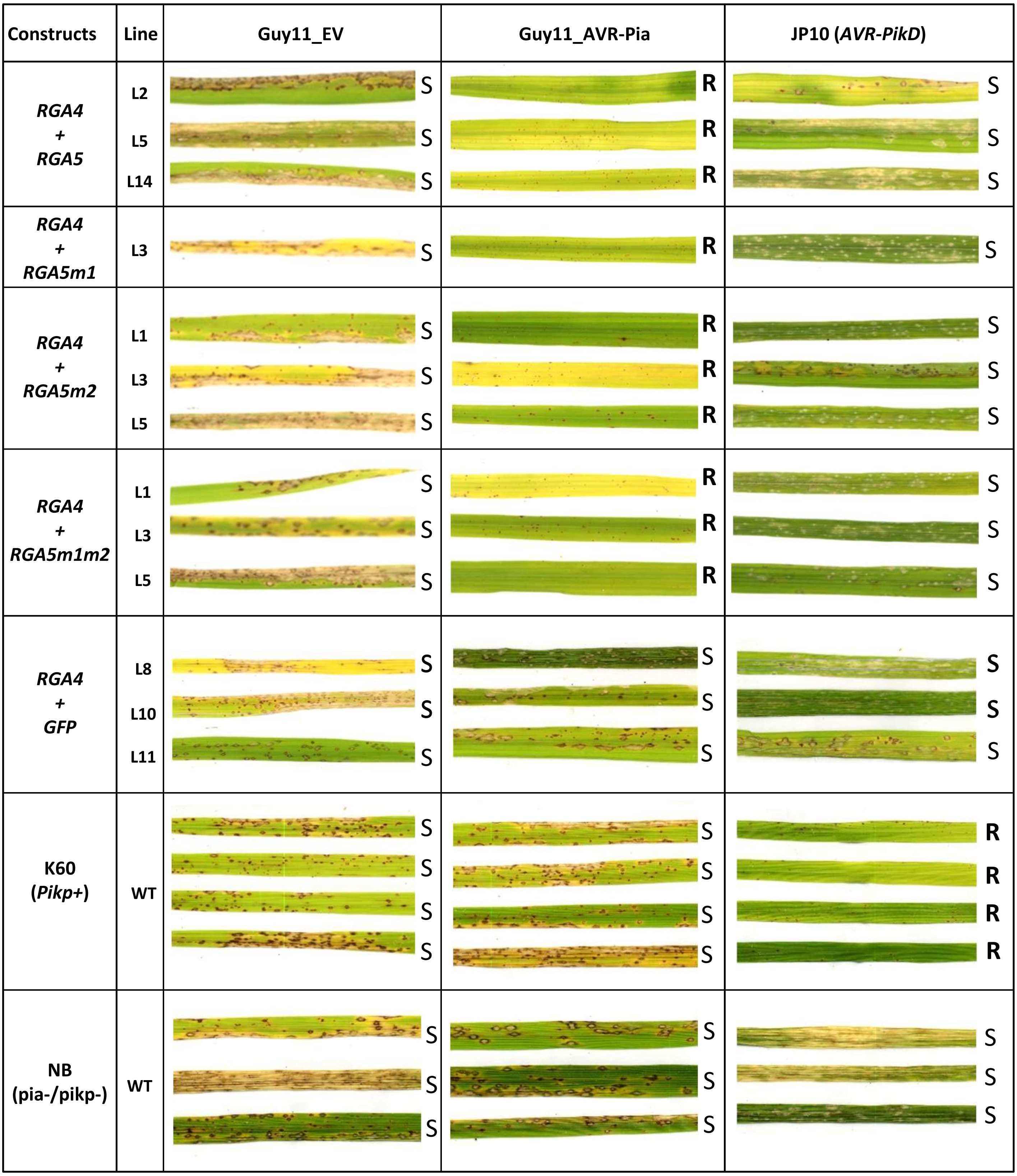

Supplemental figure 9: Inoculation of T0 transgenic plants with *M. oryzae*. The rice cultivar Nipponbare was co-transformed with a genomic construct for *RGA4* and a genomic construct for *RGA5*, *RGA5m1*, *RGA5m2* or *RGA5m1m2*. A transgenic line carrying *RGA4* and the *GFP* was also generated as a control. T0 plants of the transgenic lines were spray inoculated with the transgenic strain Guy11-AVR-Pia or the wild-type JP10 (*AVR-PikD*+) isolate. The rice cultivar K60 carrying the *Pikp* resistance was used as a control for AVR-PikD specific recognition while Nipponbare (*pikp-/pia-*) served as negative control. Pictures show representative symptoms at 7 days after inoculation. Individual leaves indicate independent T1 transgenic lines (see Supplemental Table 3). S = susceptible, R = resistant.

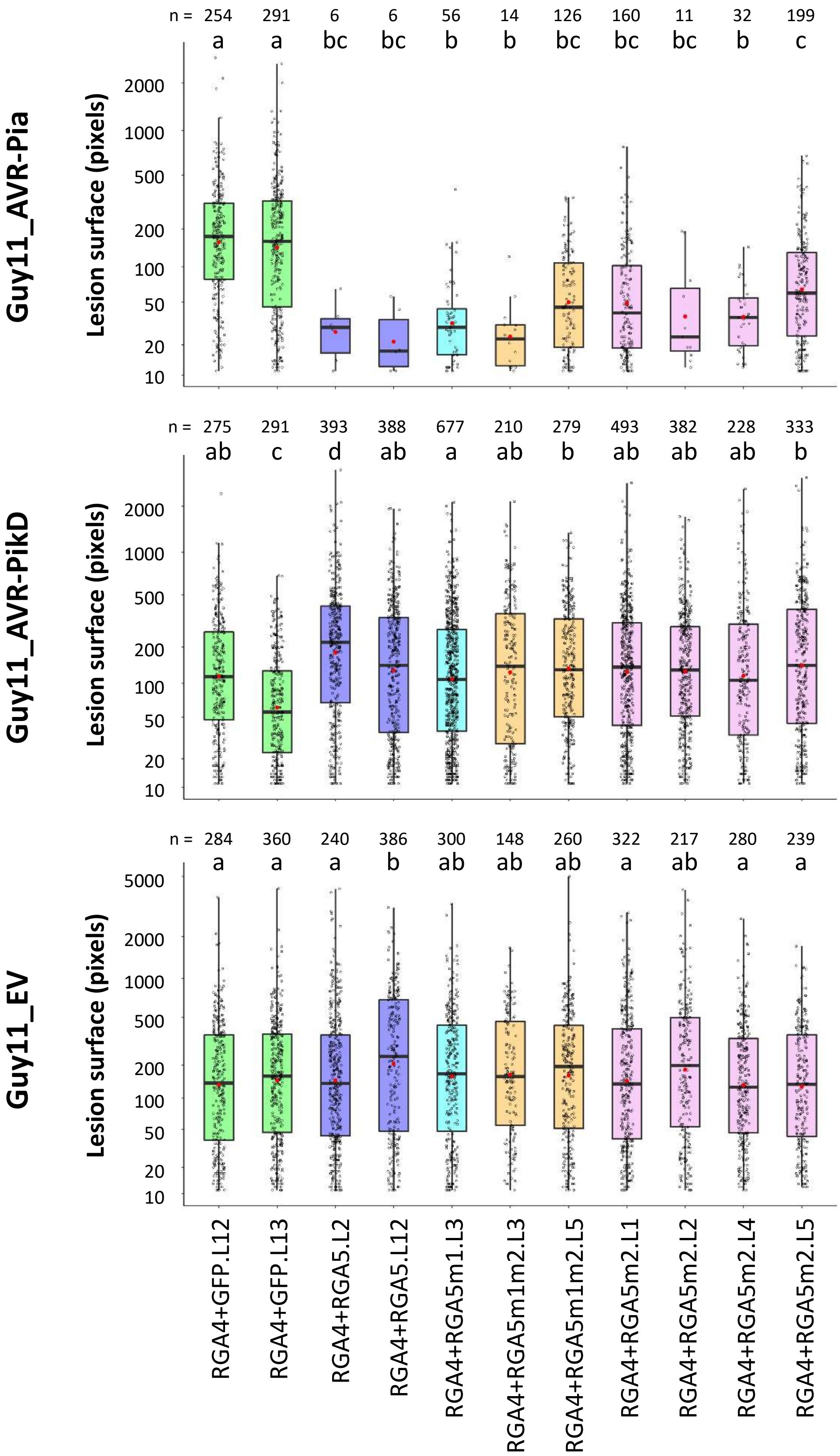

Supplemental figure 10: Disease lesion measurements after inoculation of T2 transgenic plants. Transgenic *M. oryzae* isolates carrying *AVR-Pia*, *AVR-PikD* or the empty vector (EV), were spray-inoculated on T2 transgenic plants carrying the indicated transgenes (i.e. *RGA4+GFP*, *RGA4+RGA5, RGA4+RGA5m1, RGA4+RGA5m1m2* or *RGA4+RGA5m2*). For each combination of transgenes, the rice transgenic lines used for inoculation are indicated (see Suppl. Table 3). Leaves from 5 to 8 different plants for each transgenic line were scanned 7 days after inoculation. Areas of disease lesions were measured using LeAFtool (https://github.com/sravel/LeAFtool) and plotted. The boxes represent the first quartile, median, and third quartile. Red dots indicate the mean values. Difference of lesion areas among the transgenic lines was assessed by a Kruskall-Wallis test followed by a Dunn test. For each isolate inoculated, groups with the same letter (A to C or D) are not significantly different at level 0.01. n indicates the total number of lesions measured for each rice transgenic line over one experiment.

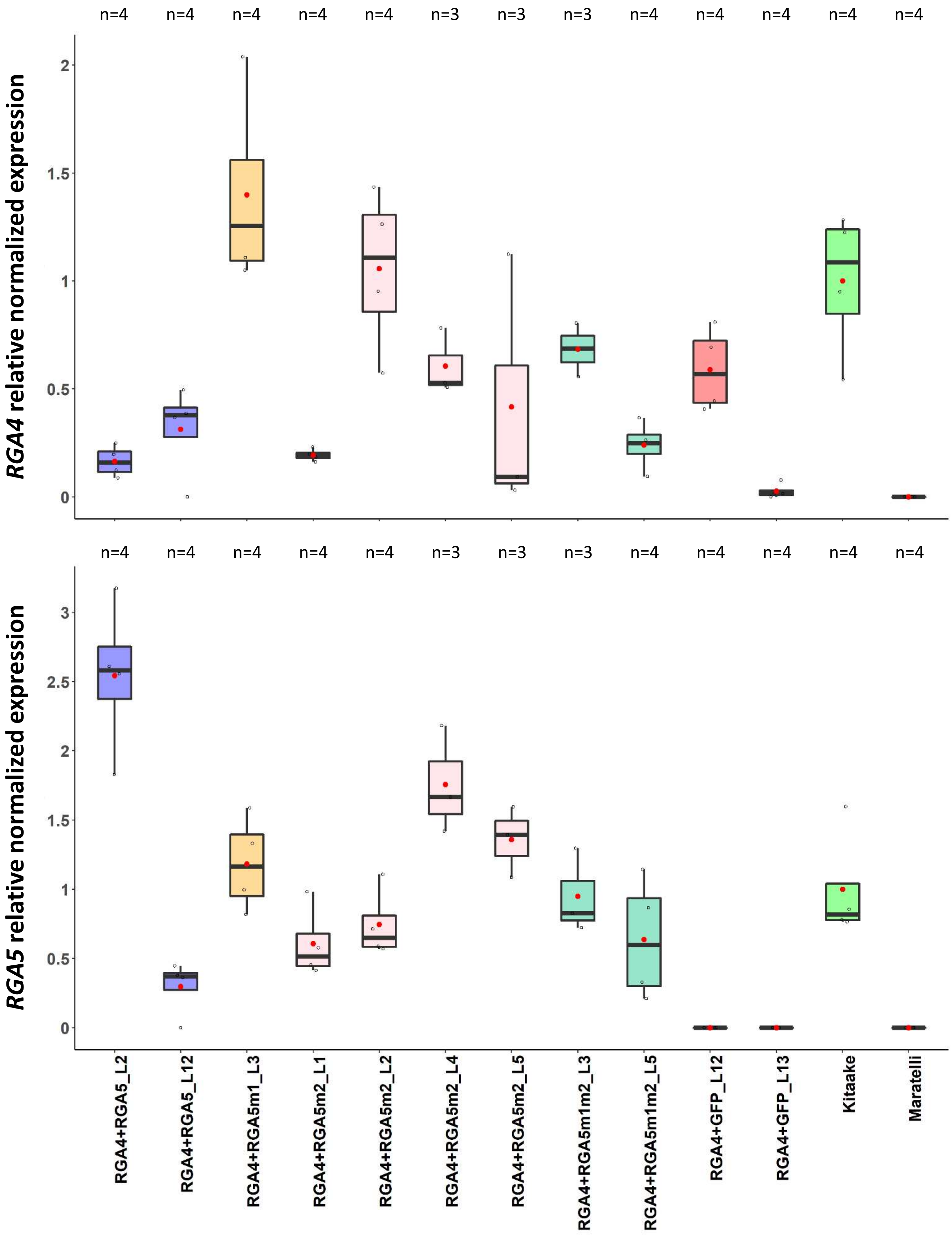

Supplemental Figure 11: Relative normalized expression of *RGA4* and *RGA5* in rice transgenic lines. mRNA levels of *RGA4* and *RGA5* in the independent rice transgenic lines (L) and in wild type Maratelli (*pia*-) and Kitaake (*Pia*+) were determined by qRT-PCR. Relative expression levels were calculated using the constitutively expressed rice actin gene (*Os03g50890*) as a reference. Relative expression of *RGA4* and *RGA5* in each transgenic line was normalized relative to the mean expression level of each gene in Kitaake. The boxes represent the first quartile, median, and third quartile. Red dots indicate the mean values calculated from n=3 or n=4 biologically independent plant pools (each pool containing RNA from 3 individual plants) for each transgenic line.

Supplemental table 1: Summary of QtPISA analysis of the minimized X-ray and model structures of MAX/HMA complexes

Complex	BE cal.mol^{-1}	ΔiG kcal.mol^{-1}	Total interface area Å^2	No. of H-Bonds	No. of Salt Bridges
**AVR1-CO39/RGA5_HMA** (PDB 5ZNG)	-6.5(-7.3)	-3.0(-4.6)	499.0(492.8)	7(6)	1(0)
**AVR-Pia/Pikp-1_HMA** (PDB 6Q76)	-8.9(-8.6)	-4.6(-4.7)	491.6(460.7)	7(7)	3(2)
**AVR-PiKD/Pikp-1_HMA** (PDB 6G10)	-10.3(-10.8)	-2.1(-2.2)	1106.6(979.3)	11(10)	9(11)
**AVR-Pia/RGA5_HMA**	-7.2	-4.3	488.0	4	3
**AVR-Pia/HMAm1m2**	-8.6	-4.3	481.4	8	2
**AVR1-CO39/HMAm1m2**	-6.8	-3.4	482.8	6	2
**AVR-PikD/HMAm1m2**	-11.3	-2.2	1052.1	13	9

As controls, the three PDB structures (5ZNG, 6Q76 and 6G10) used as templates for modelling HMA domains in complex with MAX effectors were minimized with Charmm and analysed with QtPISA. The values given between parenthesis correspond to the initial PDB structures without minimization. In the bottom section, five models of MAX/HMA complexes generated as described in the Methods were refined with Charmm and analysed with QtPISA. BE is the total Binding Energy at the interaction interface and ΔiG is the solvation energy gain. The number of H-bonds and salt-bridges formed at the complex interface are also indicated.

Supplemental Table 2: Binding and fitting parameters of AVR-PikD interaction with different HMA domains, calculated for the kinetic titrations shown in supplementary Figure 4 using the indicated interaction model for fitting SPR data.

Steady-state model	K_D (M)	R_{max} (RU)	Chi² (RU²)
AVR-PikD/RGA5_HMA wt	**8.32E-06**	861	9.51

Heterogeneous model	K_D1 (M)	k_a1 (1/Ms)	k_d1 (1/s)	K_D2 (M)	k_a2 (1/Ms)	k_d2 (1/s)	R_{max}1 (RU)	R_{max}2 (RU)	Chi² (RU²)
AVR-PikD/RGA5_HMAm1	**1.60E-09**	7.30E+05	1.17E-03	2.90E-07	6.66E+03	1.93E-03	598	521	29.4
AVR-PikD/RGA5_HMAm1m2	**5.38E-10**	8.36E+05	4.50E-04	3.60E-07	8.61E+03	3.10E-03	549	517	3.98
AVR-PikD/Pikp-1_HMA	**8.30E-10**	1.43E+06	1.19E-03	4.44E-08	3.84E+05	1.71E-02	372	119	11.9

Rice cultivar	Genes transformed	Independant transgenic line	Genotype RGA4 (T0)	Genotype RGA5 (T0)	Sequencing RGA5 PCR products (T0)	Phenotype T0 Guy11_EV	Phenotype T0 Guy11_AVR-Pia	Phenotype T0 JP10 (AVR-PikD+)	T0 seed production	Phenotype T1 Guy11_EV	Phenotype T1 Guy11_AVR-Pia	Phenotype T1 Guy11_AVR1-CO39	Phenotype T1 Guy11_AVR-PikD	Phenotype T2 Guy11_AVR-Pia	Phenotype T2 Guy11_AVR1-CO39	Phenotype T2 Guy11_AVR-PikD
Nipponbare (pia-/pikp-)	RGA4+RGA5	L1	+	+	+	S	S	S	20	nt	nt	nt	nt	nt	nt	nt
		L2	+	+	nt	S	R	S	>100	S	R	R	S	R	R	S
		L3	+	+	+	nt	nt	nt	~50	nt	nt	nt	nt	nt	nt	nt
		lacking RGA5	+	-	nt	nt	nt	nt	>100	nt	nt	nt	nt	nt	nt	nt
		L4	+	+	+	S	nt	S	5	nt	nt	nt	nt	nt	nt	nt
		L5	+	+	+	S	R	S	~50	S	R	R	S	nt	nt	nt
		L6	+	+	nt	S	nt	S	>100	nt	nt	nt	nt	nt	nt	nt
		L7	+	+	+	nt	nt	nt	10	nt	nt	nt	nt	nt	nt	nt
		lacking RGA5	+	-	nt	nt	nt	S	>100	nt	nt	nt	nt	nt	nt	nt
		L8	+	+	nt	S	S	S	>100	nt	nt	nt	nt	nt	nt	nt
		L9	+	+	nt	nt	R	nt	0	nt	nt	nt	nt	nt	nt	nt
		L10	+	+	nt	S	R	nt	0	nt	nt	nt	nt	nt	nt	nt
		L11	+	+	nt	nt	R	nt	0	nt	nt	nt	nt	nt	nt	nt
		L12	+	+	nt	S	R	nt	>100	S	R	R	S	R	R	S
		L13	+	+	nt	nt	nt	nt	0	nt	nt	nt	nt	nt	nt	nt
		L14	+	+	nt	S	R	S	10	nt	nt	nt	nt	nt	nt	nt
	RGA4+RGA5m1	L1	+	+	+	S	nt	S	>100	S	R	R	S	nt	nt	nt
		L2	+	+	+	S	S	S	>100	nt	nt	nt	nt	nt	nt	nt
		L3	+	+	+	S	R	S	~50	S	R	R	S	R	R	S
		L4	+	+	+	nt	nt	nt	0	nt	nt	nt	nt	nt	nt	nt
		L5	+	+	+	nt	nt	nt	0	nt	nt	nt	nt	nt	nt	nt
		L6	+	+	+	nt	nt	nt	0	nt	nt	nt	nt	nt	nt	nt
	RGA4+RGA5m2	L1	+	+	+	S	R	S	>100	S	R	R	S	R	R	S
		L2	+	+	+	nt	nt	nt	>100	nt	nt	nt	nt	R	R	S
		L3	+	+	+	S	R	S	9	nt	nt	nt	nt	nt	nt	nt
		L4	+	+	+	nt	nt	nt	>100	S	R	R	S	R	R	S
		L5	+	+	+	S	R	S	~50	S	R	1 dwarf plant	S	R	R	S
	RGA4+RGA5m1m2	L1	+	+	+	S	R	S	0	nt	nt	nt	nt	nt	nt	nt
		L2	+	+	+	nt	nt	nt	0	nt	nt	nt	nt	nt	nt	nt
		L3	+	+	+	S	R	S	~50	S	R	R	S	R	R	S
		L4	+	+	+	S	S	S	~50	nt	nt	nt	nt	nt	nt	nt
		L5	+	+	+	nt	R	S	4	nt	nt	nt	nt	R	R	S
		L6	+	+	+	S	S	S	~50	nt	nt	nt	nt	nt	nt	nt
	RGA4+GFP	L1	+	-	nt	nt	nt	nt	10	nt	nt	nt	nt	nt	nt	nt
		L2	+	-	nt	nt	nt	nt	10	nt	nt	nt	nt	nt	nt	nt
		L3	+	-	nt	nt	nt	nt	10	nt	nt	nt	nt	nt	nt	nt
		L4	+	-	nt	nt	nt	nt	0	nt	nt	nt	nt	nt	nt	nt
		L5	+	-	nt	nt	nt	nt	0	nt	nt	nt	nt	nt	nt	nt
		L6	+	-	nt	S	S	S	20	nt	nt	nt	nt	nt	nt	nt
		L7	+	-	nt	nt	nt	nt	10	nt	nt	nt	nt	nt	nt	nt
		L8	+	-	nt	S	S	S	>100	nt	nt	nt	nt	nt	nt	nt
		L9	+	-	nt	nt	nt	nt	0	nt	nt	nt	nt	nt	nt	nt
		L10	+	-	nt	S	S	S	>100	nt	nt	nt	nt	nt	nt	nt
		L11	+	-	nt	S	S	S	4	nt	nt	nt	nt	nt	nt	nt
		lacking RGA4	-	-	nt	nt	nt	nt	>100	nt	nt	nt	nt	nt	nt	nt
		L12	+	-	nt	S	S	S	>100	S	S	S	S	S	S	S
		lacking RGA4	-	-	nt	S	S	S	>100	nt	nt	nt	nt	nt	nt	nt
		L13	+	-	nt	nt	nt	nt	>100	S	S	S	S	S	S	S
		L14	+	-	nt	nt	nt	nt	>100	S	S	S	S	nt	nt	nt
K60 (pia-/Pikp+)	/	/	/	/	/	I	I	R	/	S	S	S	R	S	S	R
Nipponbare (pia-/pikp-)	/	/	/	/	/	S	S	S	/	S	S	S	S	S	S	S
Maratelli (pia-/pikp-)	/	/	/	/	/	S	S	S	/	S	S	S	S	S	S	S
Kanto 51 (pia-/Pik*+)	/	/	/	/	/	S	S	R	/	nt	nt	nt	nt	nt	nt	nt
Tsuyuake (pia-/Pikm+)	/	/	/	/	/	S	S	R	/	nt	nt	nt	nt	nt	nt	nt
Kitaake (Pia+/Pikp+)	/	/	/	/	/	S	R	R	/	nt	nt	nt	nt	nt	nt	nt

Supplemental Table 3: Inoculation experiments on T0, T1 and T2 rice transgenic plants. S = susceptible; R = resistant; I = intermediate resistance; nt = not tested.

Supplemental Table 4: Primers

Primers	Sequence
oCS080	GGGGACCACTTTGTACAAGAAAGCTGGGTCTCACATGGTTGAGCAAGGGTTAA
oCS108	GGGGACAAGTTTGTACAAAAAAGCAGGCTTAATGGGTGGCGGGTGGACTAACAA
oCS109	GGGGACCACTTTGTACAAGAAAGCTGGGTCTTACATAATATTGCAGCCCTCTTC
oCS118	GGGGACAAGTTTGTACAAAAAAGCAGGCTTAATGGAGCACCTTGTAAGCCTCCAG
oCS209	GGGGACAAGTTTGTACAAAAAAGCAGGCTTAATGGTGAGCAAGGGCGAGG
oCS210	GGGGACCACTTTGTACAAGAAAGCTGGGTCTTATAAGCCTGCTTTTTTGTACAAACTTG
oCS330	GTGAACGGGGTGGACAGCGTGGCGTTAGTGGGGGATCTAAGAGACAAGATCGAGGTGGTCGGCCGTGGCATTGAC
oCS331	GTCAATGCCACGGCCGACCACCTCGATCTTGTCTCTTAGATCCCCCACTAACGCCACGCTGTCCACCCCGTTCAC
oCS332	GAAATGTGGCCTCGCCGAGCTCTTGCAGGTGTCGCAGGTTGAGAAAGAGAAGACACAGCTGG
oCS333	CCAGCTGTGTCTTCTCTTTCTCAACCTGCGACACCTGCAAGAGCTCGGCGAGGCCACATTTC
oCS334	GGGGACAAGTTTGTACAAAAAAGCAGGCTTAATGGAAACGGGCAACAAATATATAGAAAAA
oCS335	GGGGACCACTTTGTACAAGAAAGCTGGGTCTTAAAAGCCGGGCCTTTTTTTC
oCS346	GGGGACAAGTTTGTACAAAAAAGCAGGCTTACTCAGAAAAACAGGGCTAAAGCAAAA
oCS347	GGGGACCACTTTGTACAAGAAAGCTGGGTCTCAATCTTTATTTGCTTGGCTGACCTGC
oCS348	GGGGACAAGTTTGTACAAAAAAGCAGGCTTAAGTGCATTAACGGGGCAACG
oCS349	GGGGACCACTTTGTACAAGAAAGCTGGGTCTCACTCTTTCTCAACTAACTCCACCATCAA
oCS351	GGGGACCACTTTGTACAAGAAAGCTGGGTCTCACTCTTTCTCAACCTGCGACACCT
oCS385	GCTTGCAGGGGTGGACAGCGTGGCGTTAGTGGGGGATCTAAGAGACAAGATCGAGGTGGTCGGCCGTGGCATTGAC
oCS386	GTCAATGCCACGGCCGACCACCTCGATCTTGTCTCTTAGATCCCCCACTAACGCCACGCTGTCCACCCCTGCAAGC
oTK017_c	AGGACCCAATCTTCAAAATGGCTAAAGTCCAAGCTACATTCGCCA
oTK036	GGAGCCTGAATGTTGAGTGGAATGATGCGGGATCAACAAGACTCATCGTCGTCA
oTK439	TATCATATGGCTGCGCCAGCTAGATCTTGCGTCTAT
oTK334	TATGGATCCCTAGTAAGGCTCGGCAGCAAG
oCS429	TGGCTGGAGGAAAGAAAGGTG
oCS430	GGTGCTGAAGGTGGATACTCTAC
oCS433	AGAGGGACCAGAACAAGTGAAC
oCS434	TGAGCTTGATTGGTCTGCAAAG
33F	GCGTGGACAAAGTTTTCAACCG
33R	TCTGGTACCCTCATCAGGCATC

Supplemental Table 5: Constructs.

Use	Final plasmid	Cloning Method	Template	Cloning primers	Backbone vector	Insert	Reference
Entry vectors for LR cloning	pSC049	/	/	/	pDONR207	dSP-AVR-Pia with start without stop (20-85)	Cesari et al., 2013
	pSC060	/	/	/	pDONR207	dSP-AVR-Pia with start with stop (20-85)	Cesari et al., 2014
	pSC120	GTW BP	pTH123.1	oCS108/oCS109	pDONR207	dSP-PWL2 with start with stop (22-145)	
	pSC129	/	/	/	pDONR207	RGA5_Cter with stop (883-1116)	Cesari et al., 2013
	pSC210	/	/	/	pDONR207	RGA5_deltaHMA with stop (1-996)	Cesari et al., 2014
	pSC309	GTW BP	pBIN19-YFP-GTW	oCS209/oCS210	pDONR207	YFP with stop	Cesari et al., 2014
	pSC467	Quikchange	pSC057	oCS330/oCS331	pDONR207	RGA5m1 with stop (1-1116)	Cesari et al., 2014
	pSC468	quikchange	pSC057	oCS332/oCS333	pDONR207	RGA5m2 with stop (1-1116)	
	pSC469	quikchange	pSC467	oCS332/oCS333	pDONR207	RGA5m1m2 with stop (1-1116)	
	pSC470	quikchange	pSC207	oCS330/oCS331	pDONR207	RGA5_HMAm1 with stop (997-1072)	
	pSC472	GTW BP	pSC469	oCS348/oCS351	pDONR207	RGA5_HMAm1m2 with stop (991-1072)	
	pSC474	GTW-BP	pTK210	oCS334/oCS335	pDONR207	dSP-AVR-PikD with stop (22-113)	
	pSC500	GTW-BP	pSC496	oCS346/oCS347	pDONR207	Pikp-1_HMA with stop (182-263)	
	pSC501	GTW-BP	pSC057	oCS348/oCS349	pDONR207	RGA5_HMA with stop (991-1072)	
	pSC502	GTW-BP	pSC469	oCS118/oCS080	pDONR207	RGA5_C-ter_m1m2 with stop (883-1116)	
	pSC629	GTW-BP	pSC467	oCS348/oCS349	pDONR207	RGA5_HMAm1 with stop (991-1072)	
	pSC630	GTW-BP	pSC468	oCS348/oCS351	pDONR207	RGA5_HMAm2 with stop (991-1072)	
	pSC631	GTW-BP	pSC467	oCS118/oCS080	pDONR207	RGA5_Cter_m1 with stop (883-1116)	
	pSC632	GTW-BP	pSC468	oCS118/oCS080	pDONR207	RGA5_Cter_m2 with stop (883-1116)	
Y2H	pSC001	/	/	/	pGBKT7-BD	BD:dSP-AVR1-CO39 (22-89)	Cesari et al., 2013
	pCV243	GTW LR	pSC060	/	pGBKT7-BD_GTW	BD:dSP-AVR-Pia (20-85)	
	pSC490	GTW LR	pSC474	/	pGBKT7-BD_GTW	BD:dSP-AVR-PikD (22-113)	
	pGBKT7-BD	GTW LR	/	/	pGBKT7-BD	BD	Clontech
	pSC548	GTW LR	pSC501	/	pGADT7-AD_GTW	AD:RGA5_HMA (991-1072)	
	pSC636	GTW LR	pSC629	/	pGADT7-AD_GTW	AD:RGA5_HMAm1 (991-1072)	
	pSC637	GTW LR	pSC630	/	pGADT7-AD_GTW	AD:RGA5_HMAm2 (991-1072)	
	pSC485	GTW LR	pSC472	/	pGADT7-AD_GTW	AD:RGA5_HMAm1m2 (991-1072)	
	pCV226	GTW LR	pSC129	/	pGADT7-AD_GTW	AD:RGA5_Cter (883-1116)	
	pSC638	GTW LR	pSC631	/	pGADT7-AD_GTW	AD:RGA5_Cter_m1 (883-1116)	
	pSC639	GTW LR	pSC632	/	pGADT7-AD_GTW	AD:RGA5_Cter_m2 (883-1116)	
	pSC549	GTW LR	pSC502	/	pGADT7-AD_GTW	AD:RGA5_Cter_m1m2 (883-1116)	
	pSC547	GTW LR	pSC500	/	pGADT7-AD_GTW	AD:Pikp-1_HMA (182-263)	
	pGADT7-AD	GTW LR	/	/	pGADT7-AD	AD	Clontech
Co-IP and HR tests in *N. benthamiana*	pSC065.3	GTW LR	pSC049	/	pBIN19-GTW-3HA	dSP_AVR-Pia:3HA (20-85)	
	pSC495.1.1	GTW LR	/	/	pCambia1300	dSP_AVR-PikD:HA (22-113)	Maqbool et al., 2015
	pSC598.1	GTW LR	/	/	pCambia1300	dSP_AVR-PikC:HA (22-113)	Maqbool et al., 2015
	pDO134	GTW LR	pSC120	/	pBIN19-3HA-GTW	3HA:PWL2 (22-145)	
	pSC545.2	GTW LR	pSC501	/	pBIN19-YFP-GTW	YFP:RGA5_HMA (991-1072)	
	pSC478.2.1	GTW LR	pSC470	/	pBIN19-YFP-GTW	YFP:RGA5_HMAm1 (997-1072)	
	pSC480.1	GTW LR	pSC472	/	pBIN19-YFP-GTW	YFP:RGA5_HMAm1m2 (991-1072)	
	pSC544.2	GTW LR	pSC500	/	pBIN19-YFP-GTW	YFP:Pikp-1_HMA (182-263)	
	pSC276	GTW LR	pSC210	/	pBIN19-YFP-GTW	YFP:RGA5_deltaHMA (1-996)	
	pSC310	GTW LR	pSC309	/	pBIN19-YFP-GTW	YFP	
	pSC078.3	/	/	/	pBIN19-YFP-GTW	YFP:RGA5 (1-1116)	Cesari et al., 2013
	pSC475	GTW LR	pSC467	/	pBIN19-YFP-GTW	YFP:RGA5m1 (1-1116)	
	pSC476	GTW LR	pSC468	/	pBIN19-YFP-GTW	YFP-RGA5m2 (1-1116)	
	pSC477.1	GTW LR	pSC469	/	pBIN19-YFP-GTW	YFP:RGA5m1m2 (1-1116)	
	pSC061	/	/	/	pBIN19-3HA-GTW	RGA4:3HA (1-996)	Cesari et al., 2013
	pSC095	/	/	/	pBIN19-GTW-3HA	dSP_AVR-Pia (20-85)	Cesari et al., 2014
	pSC497	/	/	/	pICSL4723	Pikp-1:Hellfire/Pikp-2:HA	provided by Mark Banfield
Magnaporthe oryzae transformation	pCV10	yeast gap repair	genomic DNA of *M. oryzae* IN03	oTK017_c/oTK036	pDL02	pRP27::SP-AVR1-CO39 (1-89)	(identical to pCB027 from Ribot et al, 2013)
	pCB1004-pex22	/	/	/	pCB1004	pAVR-Pia::SP-AVR-Pia (1-85)	Yoshida et al., 2009
	pCB1004-pex31	/	/	/	pCB1004	pAVR-PikD::SP-AVR-PikD (1-113)	Yoshida et al., 2009
	pCB1004-EV	/	/	/	pCB1004	Empty vector	Yoshida et al., 2009
Rice stable transgenics	pSC038	/	/	/	pCambia1300	pRGA4::RGA4genomic	Cesari et al., 2013
	pSC086	/	/	/	pCambia2300	pRGA5::RGA5genomic	Cesari et al., 2013
	pADu52.3	Quikchange	pAHC17-pRGA5::RGA5	oSC385/oSC386	pCambia2300	pRGA5::RGA5m1	Okuyama et al., 2011
	pADu50.3	Quikchange	pAHC17-pRGA5::RGA5	oSC332/oSC333	pCambia2300	pRGA5::RGA5m2	Okuyama et al., 2011
	pADu51.3	Quikchange	pADu52.3	oSC332/oSC333	pCambia2300	pRGA5::RGA5m1m2	
SPR	pCV64	/	/	/	pET15b	dSP-AVR-Pia	de Guillen et al., 2015
	pCV61.2	/	/	/	pET15b	dSP-AVR1-CO39	de Guillen et al., 2015
	pLM004	in fusion	synthetic gene	/	pDB_ccdb_his_3C	dSP-AVR-PikD	
	pLM003	in fusion	synthetic gene	/	pDb_ccdb_pepL_his_3C	dSP-AVR1-CO39 T41G	
	pCV147	recombination	pSC60	oTK439/oTK334	pET15b	dSP-AVR-Pia F24S	Ortiz et al., 2017
	pLM012	in fusion	synthetic gene	/	pDB_ccdb_his_MBP_3C	MBP:RGA5_HMA	
	pLM015	in fusion	synthetic gene	/	pDB_ccdb_his_MBP_3C	MBP:RGA5_HMAm1	
	pLM018	in fusion	synthetic gene	/	pDB_ccdb_his_MBP_3C	MBP:RGA5_HMAm1m2	
	pLM009	in fusion	synthetic gene	/	pDB_ccdb_his_MBP_3C	MBP:Pikp-1_HMA	
