# Supplementary material for: New recognition specificity in a plant immune receptor by molecular engineering of its integrated domain
Source: Nat Commun. 2022 Mar 21;13:1524. doi: 10.1038/s41467-022-29196-6 (PMC8938504; doi:10.1038/s41467-022-29196-6)
Supplement: Supplementary file 3 — Reporting Summary [file 41467_2022_29196_MOESM3_ESM.pdf]

nature portfolio

Corresponding author(s): Thomas Kroj, Stella Cesari

Last updated by author(s): 27/01/2022

Reporting Summary

Nature Portfolio wishes to improve the reproducibility of the work that we publish. This form provides structure for consistency and transparency in reporting. For further information on Nature Portfolio policies, see our Editorial Policies and the Editorial Policy Checklist.

Statistics

For all statistical analyses, confirm that the following items are present in the figure legend, table legend, main text, or Methods section.

n/a	Confirmed	
☐	☒	The exact sample size (*n*) for each experimental group/condition, given as a discrete number and unit of measurement
☐	☒	A statement on whether measurements were taken from distinct samples or whether the same sample was measured repeatedly
☐	☒	The statistical test(s) used AND whether they are one- or two-sided<br>*Only common tests should be described solely by name; describe more complex techniques in the Methods section.*
☐	☒	A description of all covariates tested
☒	☐	A description of any assumptions or corrections, such as tests of normality and adjustment for multiple comparisons
☒	☐	A full description of the statistical parameters including central tendency (e.g. means) or other basic estimates (e.g. regression coefficient) AND variation (e.g. standard deviation) or associated estimates of uncertainty (e.g. confidence intervals)
☒	☐	For null hypothesis testing, the test statistic (e.g. *F*, *t*, *r*) with confidence intervals, effect sizes, degrees of freedom and *P* value noted<br>*Give P values as exact values whenever suitable.*
☒	☐	For Bayesian analysis, information on the choice of priors and Markov chain Monte Carlo settings
☒	☐	For hierarchical and complex designs, identification of the appropriate level for tests and full reporting of outcomes
☒	☐	Estimates of effect sizes (e.g. Cohen's *d*, Pearson's *r*), indicating how they were calculated

Our web collection on statistics for biologists contains articles on many of the points above.

Software and code

Policy information about availability of computer code

Data collection	LeAFtool (open source https://github.com/sravel/LeAFtool), Lightcycler 480 Software (Roche, version 1.5) , Biacore T200 BiaEvaluation software 3.0 (GE Healthcare)
Data analysis	QtPISA (open source), CHARM (open source), R (open source), PyMOL (Schrödinger)

For manuscripts utilizing custom algorithms or software that are central to the research but not yet described in published literature, software must be made available to editors and reviewers. We strongly encourage code deposition in a community repository (e.g. GitHub). See the Nature Portfolio guidelines for submitting code & software for further information.

Data

Policy information about availability of data

All manuscripts must include a data availability statement. This statement should provide the following information, where applicable:

- Accession codes, unique identifiers, or web links for publicly available datasets
- A description of any restrictions on data availability
- For clinical datasets or third party data, please ensure that the statement adheres to our policy

All data generated or analysed during this study are included in the manuscript (and its supplementary information files) or are available from the corresponding authors on reasonable request.

Field-specific reporting

Please select the one below that is the best fit for your research. If you are not sure, read the appropriate sections before making your selection.

☒ Life sciences ☐ Behavioural & social sciences ☐ Ecological, evolutionary & environmental sciences

For a reference copy of the document with all sections, see nature.com/documents/nr-reporting-summary-flat.pdf

Life sciences study design

All studies must disclose on these points even when the disclosure is negative.

Sample size	Sample sizes were determined according to good practice in the research field.
Data exclusions	No data were excluded from the study
Replication	All experiments were performed at least twice and numbers of replications are provided in the figure legends
Randomization	Randomisation does not apply to the type of functional experiments performed in this study.
Blinding	Blinding does not apply to the type of functional experiments performed in this study.

Reporting for specific materials, systems and methods

We require information from authors about some types of materials, experimental systems and methods used in many studies. Here, indicate whether each material, system or method listed is relevant to your study. If you are not sure if a list item applies to your research, read the appropriate section before selecting a response.

Materials & experimental systems

n/a	Involved in the study	
☐	☒	Antibodies
☒	☐	Eukaryotic cell lines
☒	☐	Palaeontology and archaeology
☒	☐	Animals and other organisms
☒	☐	Human research participants
☒	☐	Clinical data
☒	☐	Dual use research of concern

Methods

n/a	Involved in the study	
☒	☐	ChIP-seq
☒	☐	Flow cytometry
☒	☐	MRI-based neuroimaging

Antibodies

Antibodies used	anti-HA antibodies conjugated with horseradish peroxidase - Roche #12013819001, anti-HA high affinity IgG1 rom rat , clone BMG 3F10, dilution 1/1000,<br>anti-Myc antibodies conjugated with horseradish peroxidase - Sigma #SAB4200742, anti-c-myc antibody from mouse, clone 9E10, dilution 1/1000,<br>anti-GFP antibodies - Roche #11814460001, anti GFP IgG1K from mouse , clones 7.1 and 13.1, dilution 1/1000<br>anti-mouse antibodies conjugated with horseradish peroxidase - Sigma #A4416, anti-mouse IgG antibody produced in goat, dilution 1/10000
Validation	only commercial antibodies were used
